# Facile synthesis of 4*H*-chromene derivatives via base-mediated annulation of *ortho*-hydroxychalcones and 2-bromoallyl sulfones

**DOI:** 10.3762/bjoc.12.3

**Published:** 2016-01-06

**Authors:** Srinivas Thadkapally, Athira C Kunjachan, Rajeev S Menon

**Affiliations:** 1 Medicinal Chemistry and Pharmacology Division, CSIR-Indian Institute of Chemical Technology, Hyderabad 500 007, India

**Keywords:** allenes, chromenes, cyclocondenzation, sulfones, vinylic substitution

## Abstract

The cesium carbonate-mediated reaction of 2-bromoallyl sulfones and *ortho*-hydroxychalcones furnished 3-arylsulfonyl-4*H*-chromene derivatives in 58–67% yield (18 examples). 2-Bromoallyl sulfones functioned as synthetic surrogates for allenyl sulfones in the reaction.

## Findings

**Figure 1:**
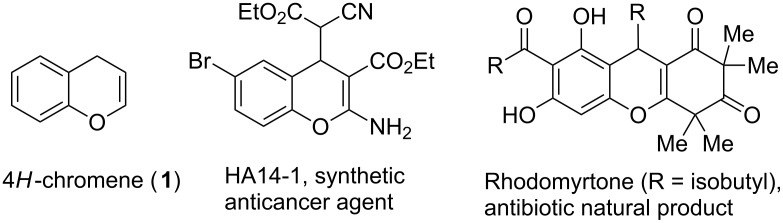
4*H*-chromene (**1**) and some of its biologically active derivatives.

Benzo[*b*]dihydropyran, commonly known as 4*H*-chromene (**1**), is a privileged heterocyclic scaffold that is found in a variety of biologically active natural and synthetic products (Figure 1) [1–3]. For example, the synthetic chromene derivative HA14-1 (Figure 1) has been shown to bind to the cellular protein Bcl-2 and to induce apoptotic cell death [4]. The natural chromene rhodomyrtone (Figure 1) is known to exhibit potent antibacterial activity [5]. As a consequence, a number of methods have been developed for the synthesis of substituted 4*H*-chromenes [6]. This includes, inter alia, transition metal-mediated cyclizations [7], multicomponent reactions [8], ring-closing metathesis approaches [9–10], tandem reactions of 1,3-dicarbonyl compounds [11–12] and cyclocondenzation reactions of salicylic aldehydes with α,β-unsaturated carbonyl compounds [13–15]. The utility of some of these methods are limited by drawbacks such as lengthy substrate synthesis, high cost of catalysts and tedious procedures. Therefore, general synthetic methods for accessing substituted chromene derivatives from readily available materials are still in demand.

**Scheme 1:**
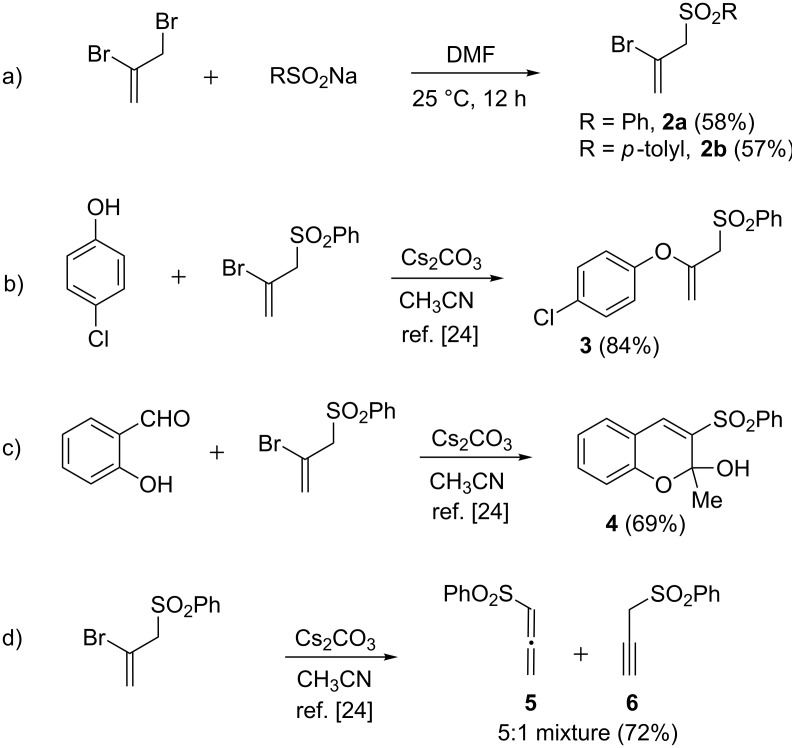
a) Preparation of 2-bromoallyl sulfones **2a**,**b**; b) reaction of **2a** with 4-chlorophenol and Cs_2_CO_3_; c) reaction of **2a** with salicylaldehyde and Cs_2_CO_3_ and d) reaction of **2a** with Cs_2_CO_3_.

During the course of our recent investigations on annulation reactions of unsaturated sulfones [16–17], we became interested in the possibility of exploiting allenyl sulfones as a building block for heterocyclic sulfones. The synthetic potential of allenyl sulfones remains largely unexploited. This is in sharp contrast with the widespread use of electronically similar allenyl esters (allenoates) in numerous useful reactions (see for examples [18–20]). The propensity of allenyl sulfones to oligomerise and display anomalous reactivity profiles in presence of base has, to some extent, dissuaded chemists from devising synthetic applications of allenyl sulfones [21–22]. We envisaged that such problems may be circumvented by developing a synthetic surrogate for the sensitive allenyl sulfones. Investigations along this direction led to the discovery that the easily prepared 2-bromoallyl sulfones **2a**,**b** function as allenyl sulfone surrogates in the presence of cesium carbonate (Scheme 1, path a). Bromoallyl sulfones **2a**,**b** partake in a cesium carbonate-mediated formal vinylic substitution reaction with heteronucleophiles to afford valuable multifunctional building blocks [23]. For example, the reaction of **2a** with 4-chlorophenol afforded the enol ether **3** in 84% yield (Scheme 1, path b) [24]. Similarly, treatment of **2a** with salicylaldehyde furnished the 3-sulfonyl-2*H*-chromene derivative **4** in 69% yield (Scheme 1, path c) [24]. The formation of allenyl sulfone **5** and propargyl sulfone **6** in the reaction of **2a** with cesium carbonate indicated that **5** is an intermediate in the above-mentioned reactions (Scheme 1, path d) [24].

**Scheme 2:**
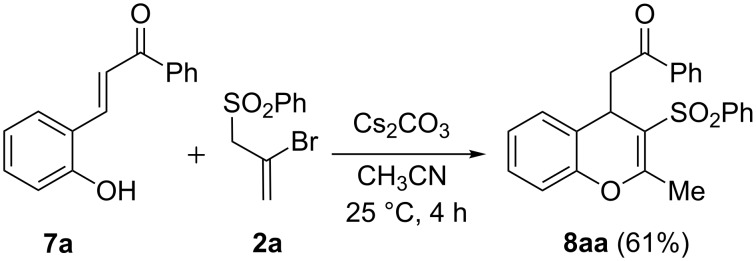
Base-mediated cyclization reaction of *o*-hydroxychalcone **7a** and 2-bromoallyl sulfone **2a**.

The facile cyclocondenzation of salicylaldehyde with **2a** (Scheme 1, path c) prompted us to explore analogous annulation reactions for the synthesis of functionalized chromene derivatives. The biological activities exhibited by many 4*H*-chromene derivatives provided an added incentive for this investigation [1]. We envisaged that the presence of a Michael acceptor double bond at the *ortho* position of a phenol would offer avenues for carbon–carbon bond forming annulation in its reaction with **2a**,**b**. In view of their well-known reactivity profiles, diversity options, stability, and ease of preparation, *ortho*-hydroxychalcones were considered to be a suitable choice for this purpose. A pilot reaction between the *o*-hydroxychalcone **7a** and bromoallyl sulfone **2a** in the presence of 2 equivalents of cesium carbonate in acetonitrile afforded the 4*H*-chromene derivative **8aa** in 61% yield (Scheme 2). It may be noted that these reaction conditions were developed for the reaction of **2a** with phenols (see Scheme 1, paths b and c) [24].

In the ^1^H NMR spectrum of **8aa**, three sets of doublet of doublets were visible at δ 4.52 (1H, *J* = 2.3 and 9.0 Hz), δ 3.58 (1H, *J* = 2.3 and 17.1 Hz) and δ 3.33 (1H, *J* = 9.0 and 17.1 Hz) arising from the -CH_2_–CH- fragment. The methyl group protons resonated as a singlet at δ 2.51. A peak at δ 197.4 in the ^13^C NMR spectrum along with the absorption peak at 1680 cm^−1^ in the IR spectrum confirmed the presence of the keto group. All other signals were in agreement with the assigned structure.

**Scheme 3:**
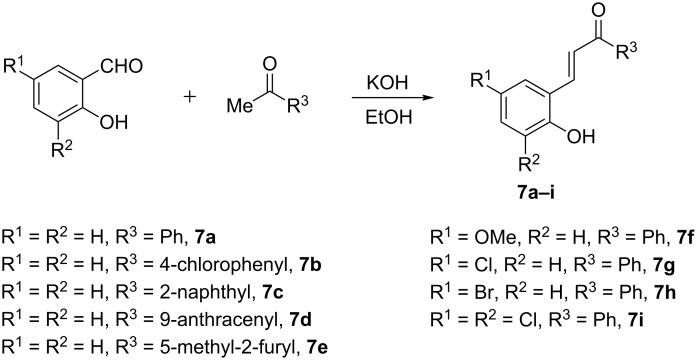
Preparation of *ortho*-hydroxychalcones **7a–i**.

In order to explore the scope and generality of this facile 4*H*-chromene synthesis, a variety of *o*-hydroxychalcones were prepared as previously described (Scheme 3) [6].

**Scheme 4:**
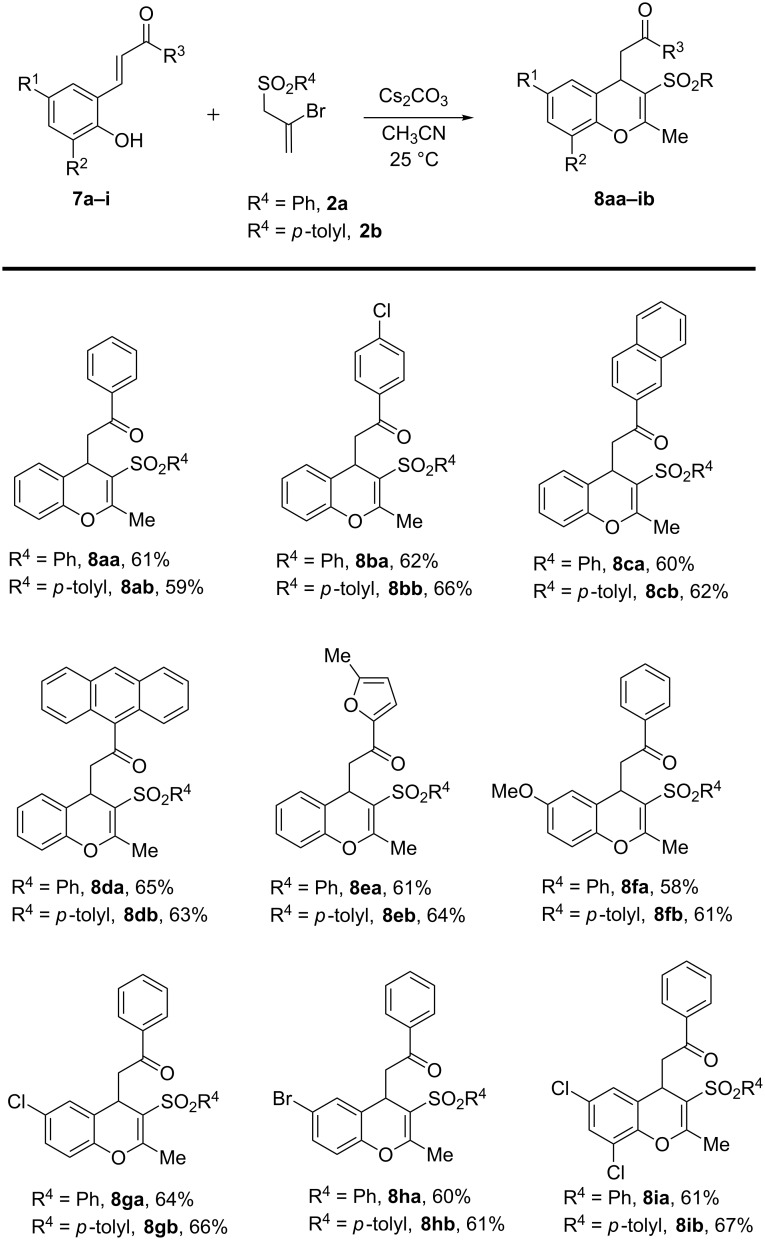
Synthesis of 4*H*-chromenes via base-mediated reactions of **7a–i** and **2a,b**. Reaction conditions: **7a–i** (0.25 mmol), **2a,b** (0.30 mmol), Cs_2_CO_3_ (0.60 mmol), CH_3_CN (3 mL), 25 °C, 4 h. Yields of isolated products are shown.

The cesium carbonate-mediated reaction of 2-bromoallyl sulfones **2a**,**b** with *o*-hydroxychalcones **7a**–**i** proceeded uneventfully to afford the corresponding 2-methyl-3-arylsulfonyl-4*H*-chromene derivatives **8aa–8ib** (Scheme 4).

The annulation reaction appears to be general as evident from the results in Scheme 4. The chalcone component can accommodate chloro, bromo and methoxy groups as aromatic substituents. Polycyclic aromatic hydrocarbon frameworks (naphthalene and anthracene rings) as well as a representative heterocyclic ring (furan) may be incorporated into the 4*H*-chromene skeleton product by using chalcones (**7c**, **7d**, and **7e**, respectively) functionalized with these moieties. Disappointingly, attempts to extend the annulation reaction to phenols with other Michael acceptors at the *ortho*-position (such as unsaturated esters, enals and nitroolefins) were not successful. Additionally, a very low yield (ca. 10%) of the product **8aa** was obtained when the chalcone formation (**7a**) and its annulation reaction with **2a** were combined into a one-pot operation (mediated by KOH in ethanol).

**Scheme 5:**
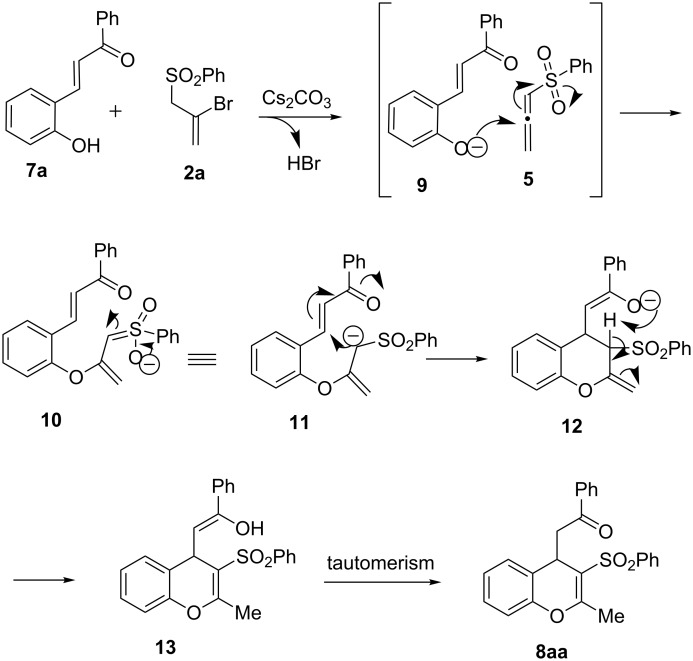
A plausible mechanistic rationalization for the formation of 4*H*-chromene derivative **8aa** from **7a** and **2a**.

A plausible mechanistic rationalization of the 4*H*-chromene formation is presented in Scheme 5. Cesium carbonate mediates the dehydrobromination of **2a** to produce the allenyl sulfone **5** (see Scheme 1, path d). Additionally, deprotonation of **7a** by Cs_2_CO_3_ generates the phenoxide anion **9**. A hetero-Michael addition of **5** and **9** results in the formation of a stabilized carbanion which may be represented as the resonance structures **10** or **11**. The α-sulfonyl carbanion **11** then undergoes an intramolecular Michael addition to the β-carbon of the enone unit to afford the enolate **12**. Isomerization of the exocyclic olefin moiety of **12** into the endocyclic position may be assisted by internal proton transfer. Tautomerization of the resultant enol **13** to its keto form affords the final product **8aa**. It may be noted that the key carbon–carbon bond forming event (conversion of **11** to **12**) here is completely regioselective as the Michael addition of the stabilized carbanion **11** occurs selectively at the α-sulfonyl position (not at the less hindered terminal of the allylic carbanion **11**).

## Conclusion

In conclusion, a base-mediated, facile synthesis of 3-sulfonyl-4*H*-chromenes from *o*-hydroxychalcones and 2-bromoallyl sulfones was developed. The starting materials are easily available and the reaction conditions are mild. 2-Bromoallyl sulfones **2a**,**b** functions as stable surrogates for the sensitive allenyl sulfones in this reaction. Functionalities such as carbonyl and sulfonyl groups are easily incorporated into the privileged scaffold of 4*H*-chromene via this method.

## Supporting Information

File 1 Experimental part and NMR spectra of synthesized compounds.
