# Supplementary material for: Facile synthesis of 4H-chromene derivatives via base-mediated annulation of ortho-hydroxychalcones and 2-bromoallyl sulfones
Source: Beilstein J Org Chem. 2016 Jan 6;12:16–21. doi: 10.3762/bjoc.12.3 (PMC4734429; doi:10.3762/bjoc.12.3)

Supporting Information

for

Facile synthesis of 4*H*-chromene derivatives via base-mediated annulation of *ortho*-hydroxychalcones and 2-bromoallyl sulfones

Srinivas Thadkapally, Athira C. Kunjachan and Rajeev S. Menon*

Address: Medicinal Chemistry and Pharmacology Division, CSIR-Indian Institute of Chemical Technology, Hyderabad 500 007, India

Email: Rajeev S. Menon - rajeevmenon@iict.res.in

*Corresponding author

Experimental part and NMR spectra of synthesized compounds

Contents	Page
General information	s1
Spectroscopic data for chalcone **7d**	s2
General procedure for the synthesis of 4*H*-chromene derivatives	s2
Spectroscopic data for 4*H*-chromene derivatives	s3
^{1}H and ^{13}C NMR spectra	s8

General information

All ^{1}H NMR and ^{13}C NMR spectra were recorded in $CDCl_3$ solvent on Varian/Bruker 300 MHz, a Varian Unity 400 MHz and Avance 500 MHz spectrometer at ambient temperature. IR spectra were recorded on Nicolet 380 FTIR spectrophotometer. Mass spectra were obtained on a Finnegan Mat1020B, a micromass VG 70-70H or an Agilent technologies LC/MSD treapSL spectrometer operating at 70eV using the direct inlet system and high resolution mass spectra (HRMS) were recorded on a QSTAR XL Hybrid MS/MS mass spectrometer. Melting points were recorded on an electrothermal apparatus and are uncorrected. Technical grade ethyl acetate and petroleum ether used for column chromatography were distilled prior to use. Column chromatography was carried out using silica gel (60–120 mesh and 100–200 mesh) packed in glass columns. All reactions were performed in oven-dried glassware with magnetic stirring. Anhydrous acetonitrile was prepared from locally purchased LR grade solvent by standard method. Sodium benzenesulfinate, sodium *p*-toluenesulfinate, cesium carbonate, 2,3-dibromopropene, substituted salicylaldehydes and substituted acetophenones were purchased

from Sigma Aldrich and were used as received. 2-Bromoallyl sulfones **2a**,**b** [1] and *o*-hydroxychalcones[2] **7a–i** were prepared as previously described. Chalcone **7d** is a new compound and was characterised by spectroscopic analysis.

Spectroscopic data for chalcone 7d

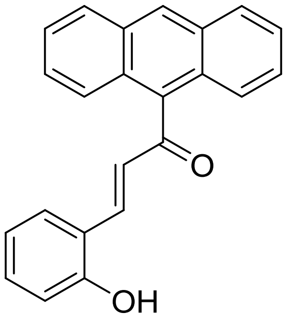

7d

(*E*)-1-(Anthracen-9-yl)-3-(2-hydroxyphenyl)prop-2-en-1-one (**7d**).

Yellow solid; 58%
R_f = 0.3 (40% ethyl acetate in hexane)
Melting point: 220-222 °C (CH_2Cl_2-hexane)
IR ν_{max} (KBr): 3050, 2360, 1610, 1574, 1451, 1363, 1293, 1265, 1244, 1201, 1188, 1150, 1113, 990, 970, 890 cm^{-1}
^{1}H NMR (300 MHz, $CDCl_3$+DMSO) δ 9.71 (s, 1H), 8.56 (s, 1H), 8.07 (d, *J* = 7.3 Hz, 1H), 7.89 (d, *J* = 8.5 Hz, 1H), 7.56-7.38 (m, 7H), 7.20-7.15 (m, 1H), 6.85-6.76 (m, 2H);
^{13}C NMR (75 MHz, $CDCl_3$+DMSO) δ 200.2, 157.0, 144.1, 134.7, 131.8, 130.6, 128.9, 128.4, 128.1, 127.8, 127.5, 125.9, 125.0, 124.9, 120.7, 119.1, 116.1;
HRMS (ESI) calcd for $C_{23}H_{16}O_2Na$ (M+Na) 347.1048; found 347.1035.

General procedure for the synthesis of 4*H*-chromene derivatives

Cesium carbonate (0.60 mmol) was added to a solution of *ortho*-hydroxychalcone **7a–i** (0.25 mmol) and 2-bromoallyl sulfone **2a**,**b** (0.3 mmol) in anhydrous acetonitrile (3 mL). The resulting solution was stirred at ambient temperature for 4 h. The reaction mixture was then partitioned between dichloromethane and ice cold water, and aqueous phase was extracted with ethyl acetate. The combined organic phases were dried with anhydrous sodium sulfate and concentrated under reduced pressure. Column chromatography on silica gel using petroleum ether–ethyl acetate as eluent afforded analytically pure samples of 4*H*-chromene products **8aa–8ib**.

[1] Undeela, S.; Thadkapally, S.; Nanubolu, J. B.; Singarapu, K. K.; Menon, R. S. *Chem. Commun*. **2015**, *51*, 13748.
[2] Yin, G.; Fan, L.; Ren, T.; Zheng, C.; Tao, Q.; Wu, A.; She, N. *Org. Biomol. Chem*. **2012**, *10*, 8877.

Spectroscopic data for 4*H*-chromene derivatives 8aa–ib

2-[2-Methyl-3-(phenylsulfonyl)-*4H*-chromen-4-yl]-1-phenylethanone (**8aa**). Compound **8aa** (62 mg, 61%) was obtained as a white solid; R_f = 0.7 (30% ethyl acetate in hexane); Mp. 122-124 °C (CH_2Cl_2-hexane); IR ν_{max} (KBr): 3444, 3063, 2924, 1680, 1639, 1487, 1384, 1315, 1276, 1177, 1070, 998.21, 776, 623 cm^{-1}; ^{1}H NMR (400 MHz, $CDCl_3$) δ: 7.92-7.87 (m, 4H), 7.56-7.47 (m, 4H), 7.41 (t, *J* = 7.6 Hz, 2H), 7.20 (d, *J* = 7.5 Hz, 1H), 7.13 (td, *J* = 7.8, 1.6 Hz, 1H), 6.99-6.95 (m, 2H), 4.52 (dd, J = 9.0, 2.3 Hz, 1H), 3.58 (dd, *J* = 17.1, 2.3 Hz, 1H), 3.33 (dd, *J* = 17.1, 9.0 Hz, 1H), 2.51 (s, 3H); ^{13}C NMR (125 MHz, $CDCl_3$, 2 signals are not observed in spectrum possibly due to overlapping signals) δ 197.4, 161.4, 150.3, 141.8, 136.7, 133.1, 129.2, 128.7, 128.5, 128.1, 128.0, 127.0, 124.79, 124.5, 116.1, 115.0, 48.3, 31.8, 18.7; HRMS (ESI) calcd for $C_{24}H_{20}O_4SNa$ (M+Na) 427.0980; found 427.0975.

2-(2-Methyl-3-tosyl-4*H*-chromen-4-yl)-1-phenylethanone (**8ab**). Compound **8ab** (62 mg, 59%) was obtained as a white solid; R_f = 0.6 (30% ethyl acetate in hexane); Mp. 129-131 °C (CH_2Cl_2-hexane); IR ν_{max} (KBr): 3448, 3066, 2920, 1683, 1636, 1596, 1484, 1352, 1301, 1146, 1090, 771, 749, 676, 582 cm^{-1}; ^{1}H NMR (400 MHz, $CDCl_3$) δ: 7.89-7.86 (m, 2H), 7.78 (d, *J* = 8.3 Hz, 2H), 7.54-7.50 (m, 1H), 7.43-7.39 (m, 2H), 7.28-7.26 (m, 1H), 7.21-7.19 (m, 2H), 7.12 (td, *J* = 7.7, 1.6 Hz, 1H), 7.00-6.95 (m, 2H), 4.51 (dd, *J* = 9.1, 2.4 Hz, 1H), 3.56 (dd, *J* = 17.1, 2.4 Hz, 1H), 3.31 (dd, *J* = 17.1, 9.1 Hz, 1H), 2.51 (s, 3H), 2.37 (s, 3H); ^{13}C NMR (100 MHz, $CDCl_3$) δ 197.4, 161.0, 150.3, 144.1, 138.9, 136.7, 133.1, 129.8, 128.7, 128.5, 128.1, 127.9, 127.1, 124.7, 124.6, 116.0, 115.3, 48.3, 31.7, 21.5, 18.7; **HRMS** calcd for $C_{25}H_{22}O_4SNa$ (M+Na) 441.1136; found 441.1111.

1-(4-Chlorophenyl)-2-[2-methyl-3-(phenylsulfonyl)-4*H*-chromen-4-yl]ethanone (**8ba**). Compound **8ba** (68 mg, 62%) was obtained as a white solid; R_f = 0.7 (30% ethyl acetate in hexane); Mp. 137-139 °C (CH_2Cl_2-hexane); IR ν_{max} (KBr): 3448, 3063, 2925, 2852, 1684, 1637, 1487, 1402, 1394, 1356, 1318, 1282, 1198, 1127, 1053, 977, 774, 738 cm^{-1}; ^{1}H NMR (400 MHz, $CDCl_3$) δ: 7.90 (d, *J* = 7.3 Hz, 2H), 7.83 (d, *J* = 8.6 Hz, 2H), 7.56-7.55 (m, 1H), 7.52-7.48 (m, 2H), 7.39 (d, *J* = 8.6 Hz, 2H), 7.18-7.13 (m, 2H), 7.00-6.96 (m, 2H), 4.49 (dd, *J* = 8.9, 2.4 Hz, 1H), 3.55 (dd, *J* = 17.0, 2.4 Hz, 1H), 3.29 (dd, *J* = 17.0, 8.9 Hz, 1H), 2.50 (s, 3H); ^{13}C NMR (100 MHz, $CDCl_3$) δ 196.2, 161.5, 150.2, 141.8, 139.6, 135.0, 133.2, 129.6, 129.2, 128.8, 128.6, 128.0, 127.0, 124.9, 124.3, 116.1, 114.9, 48.3, 31.9, 18.7; HRMS (ESI) calcd for $C_{24}H_{19}ClO_4SNa$ (M+Na) 461.0590; found 461.0573.

1-(4-chlorophenyl)-2-(2-methyl-3-tosyl-4*H*-chromen-4-yl)ethanone (**8bb**). Compound **8bb** (75 mg, 66%) was obtained as a white solid; R_f = 0.7 (30% ethyl acetate in hexane); Mp. 144-146 °C (CH_2Cl_2-hexane); IR ν_{max} (KBr): 3448, 2924, 1682, 1654, 1635, 1586, 1488, 1461, 1282, 1205, 1090, 978, 815, 771 cm^{-1}; ^{1}H NMR (400 MHz, $CDCl_3$) δ 7.82 (d, *J* = 8.6 Hz, 2H), 7.77 (d, *J* = 8.3 Hz, 2H), 7.39 (d, *J* = 8.6 Hz, 2H), 7.29-7.26 (m, 2H), 7.17-7.12 (m, 2H), 7.00-6.95 (m, 2H), 4.48 (dd, *J* = 9.0, 2.4 Hz, 1H), 3.54 (dd, *J* = 17.0, 2.4 Hz, 1H), 3.27 (dd, *J* = 17.0, 9.0 Hz, 1H), 2.50 (s, 3H), 2.38 (s, 3H); ^{13}C NMR (100 MHz, $CDCl_3$) δ 196.3, 161.1, 150.3, 144.1, 139.6,

138.9, 135.1, 129.8, 129.6, 128.8, 128.6, 128.02, 127.0, 124.8, 124.4, 116.1, 115.1, 48.3, 31.9, 21.5, 18.7; HRMS calcd for $C_{25}H_{21}ClO_4SNa$ (M+Na) 475.0747; found 475.0725.

2-[2-Methyl-3-(phenylsulfonyl)-4*H*-chromen-4-yl]-1-(naphthalen-2-yl)ethanone (**8ca**). Compound **8ca** (68 mg, 60%) was obtained as a white solid; R_f = 0.6 (30% ethyl acetate in hexane); Mp. 112-114 $^{\circ}$C (CH_2Cl_2-hexane); IR ν_{max} (KBr): 3428, 3058, 2852, 2360, 1677, 1637, 1583, 1486, 1458, 1381, 1282, 1179, 1087, 996, 941cm^{-1}; ^{1}H NMR (500 MHz, $CDCl_3$) δ 8.43 (s, 1H), 7.98-7.92 (m, 4H), 7.86-7.84 (m, 2H), 7.60-7.47 (m, 5H), 7.20 (d, *J* = 7.6 Hz, 1H), 7.14-7.11 (m, 1H), 6.99-6.96 (m, 2H), 4.58 (dd, *J* = 9.1, 2.3 Hz, 1H), 3.73 (dd, *J* = 16.7, 2.3 Hz, 1H), 3.44 (dd, *J* = 16.7, 9.1 Hz, 1H), 2.52 (s, 3H); ^{13}C NMR (100 MHz, $CDCl_3$) δ 197.3, 161.5, 150.3, 141.9, 135.6, 134.0, 133.1, 132.5, 130.1, 129.6, 129.2, 128.7, 128.5, 128.4, 128.0, 127.7, 127.0, 126.7, 124.8, 124.4, 123.8, 116.1, 115.1, 48.5, 32.1, 18.7; HRMS calcd for $C_{28}H_{22}O_4SNa$ (M+Na) 477.1136; found 477.1118.

2-(2-Methyl-3-tosyl-4*H*-chromen-4-yl)-1-(naphthalen-2-yl)ethanone (**8cb**). Compound **8cb** (73 mg, 62%) was obtained as a white solid; R_f = 0.7 (30% ethyl acetate in hexane);Mp. 130-132 $^{\circ}$C (CH_2Cl_2-hexane); IR ν_{max} (KBr): 3448, 3061, 2924, 1677, 1636, 1595, 1484, 1354, 1302, 1204, 1181, 1120, 1088, 817, 675, 620 cm^{-1}; ^{1}H NMR (500 MHz, $CDCl_3$) δ 8.42 (s, 1H), 7.97-7.93 (m, 2H), 7.86 (d, *J* = 8.8 Hz, 2H), 7.80 (d, *J* = 8.2 Hz, 2H), 7.60-7.52 (m, 2H), 7.27-7.25 (m, 2H), 7.20 (dd, *J* = 7.9, 1.3 Hz, 1H), 7.14-7.11 (m, 1H), 6.98-6.96 (m, 2H), 4.56 (dd, *J* = 9.1, 2.4 Hz, 1H), 3.70 (dd, *J* = 16.6, 2.4 Hz, 1H), 3.42 (dd, *J* = 16.6, 9.1 Hz, 1H), 2.52 (s, 3H), 2.34 (s, 3H); ^{13}C NMR (100 MHz, $CDCl_3$) δ 197.4, 161.1, 150.3, 144.1, 139.0, 135.5, 134.0, 132.5, 130.1, 129.8, 129.6, 128.7, 128.5, 128.3, 128.0, 127.7, 127.1, 126.7, 124.7, 124.5, 123.8, 116.1, 115.3, 48.4, 32.1, 21.5, 18.7; HRMS calcd for $C_{29}H_{24}O_4SNa$ (M+Na) 491.1293; found 491.1273.

1-(Anthracen-9-yl)-2-[2-methyl-3-(phenylsulfonyl)-4*H*-chromen-4-yl]ethanone (**8da**). Compound **8da** (82 mg, 65%) was obtained as a yellow solid; R_f = 0.6 (30% ethyl acetate in hexane); Mp. 187-189 $^{\circ}$C (CH_2Cl_2-hexane); IR ν_{max} (KBr): 3434, 3053, 1700, 1637, 1581, 1486, 1384, 1302, 1230, 1143, 1098, 734, 722, 634 cm^{-1}; ^{1}H NMR (500 MHz, $CDCl_3$) δ 8.42 (s, 1H), 8.03-8.01 (m, 2H), 7.96 (d, *J* = 8.6 Hz, 2H), 7.66-7.61 (m, 2H), 7.58-7.55 (m, 2H), 7.43-7.40 (m, 2H), 7.35-7.21 (m, 6H), 7.08 (d, *J* = 8.2 Hz, 1H), 4.82 (dd, *J* = 9.4, 2.0 Hz, 1H), 3.57 (dd, *J* = 19.3, 2.0 Hz, 1H), 3.42 (dd, *J* = 19.3, 9.4 Hz, 1H), 2.48 (s, 3H); ^{13}C NMR (125 MHz, $CDCl_3$) δ 207.5, 161.4, 150.6, 141.9, 135.4, 133.2, 130.8, 129.3, 129.1, 128.6, 128.3, 128.2, 127.1, 126.8, 126.7, 125.3, 125.0, 124.8, 123.9, 116.3, 114.9, 55.3, 31.0, 18.5; HRMS (ESI) calcd for $C_{32}H_{24}O_4SNa$ (M+Na) 527.1293; found 527.1276.

1-(Anthracen-9-yl)-2-(2-methyl-3-tosyl-4*H*-chromen-4-yl)ethanone (**8db**). Compound **8db** (82 mg, 63%) was obtained as a white solid; R_f = 0.6 (30% ethyl acetate in hexane); Mp. 211-213 $^{\circ}$C (CH_2Cl_2-hexane); IR ν_{max} (KBr): 3434, 3053, 2922, 1702, 1640, 1486, 1289, 1192, 1110, 1086, 776 cm^{-1}; ^{1}H NMR (500 MHz, $CDCl_3$) δ 8.41 (s, 1H), 7.95 (d, *J* = 8.4 Hz, 2H), 7.89 (d, *J* = 8.0 Hz, 2H), 7.65 (d, *J* = 7.3 Hz, 1H), 7.43-7.20 (m, 10H), 7.07 (d, *J* = 8.0 Hz, 1H), 4.81 (d, *J* = 9.2 Hz, 1H), 3.55 (d, *J* = 19.0 Hz, 1H), 3.41 (dd, *J* = 19.0, 9.2 Hz, 1H), 2.47 (s, 3H), 2.43 (s, 3H);

^{13}C NMR (100 MHz, $CDCl_3$, 2 signals are not observed in spectrum possibly due to overlapping signals) δ 207.5, 160.9, 150.7, 144.1, 139.1, 135.4, 130.8, 129.9, 129.2, 128.6, 128.2, 127.1, 126.8, 126.7, 125.3, 124.9, 123.9, 116.3, 115.2, 55.3, 31.0, 21.6, 18.5; HRMS (ESI) calcd for $C_{33}H_{26}O_4SNa$ (M+Na) 541.1449; found 541.1436.

2-[2-Methyl-3-(phenylsulfonyl)-4*H*-chromen-4-yl]-1-(5-methylfuran-2-yl)ethanone (**8ea**). Compound **8ea** (62 mg, 61%) was obtained as a white solid; R_f = 0.7 (30% ethyl acetate in hexane); Mp. 127-129 °C (CH_2Cl_2-hexane); IR ν_{max} (KBr): 3448, 3063, 2923, 1650, 1581, 1489, 1459, 1370, 1306, 1231, 1191, 1021, 796, 722, 650 cm^{-1}; ^1H NMR (500 MHz, $CDCl_3$) δ 7.93-7.91 (m, 2H), 7.58-7.55 (m, 1H), 7.52-7.49 (m, 2H), 7.17 (dd, *J* = 7.6, 1.4 Hz, 1H), 7.13 (td, *J* = 7.9, 1.6 Hz, 1H), 7.06 (d, *J* = 3.5 Hz, 1H), 6.99-6.95 (m, 2H), 6.08 (d, *J* = 3.5 Hz, 1H), 4.45 (dd, *J* = 9.3, 2.7 Hz, 1H), 3.36 (dd, *J* = 16.3, 2.7 Hz, 1H), 3.11 (dd, *J* = 16.3, 9.3 Hz, 1H), 2.49 (s, 3H), 2.34 (s, 3H); ^{13}C NMR (100 MHz, $CDCl_3$) δ 185.4, 161.4, 157.9, 151.2, 150.2, 141.9, 133.1, 129.2, 128.7, 127.9, 127.0, 124.8, 124.2, 119.5, 116.0, 114.9, 108.9, 47.5, 32.0, 18.7, 14.0; HRMS (ESI) calcd for $C_{23}H_{20}O_5SNa$ (M+Na) 431.0929; found 431.0904.

2-(2-Methyl-3-tosyl-4*H*-chromen-4-yl)-1-(5-methylfuran-2-yl)ethanone (**8eb**). Compound **8eb** (68 mg, 64%) was obtained as a white solid; R_f = 0.7 (30% ethyl acetate in hexane); Mp. 130-132 °C (CH_2Cl_2-hexane); IR ν_{max} (KBr): 3448, 3128, 2920, 1663, 1641, 1518, 1314, 1301, 1209, 814, 675, 617 cm^{-1}; ^1H NMR (500 MHz, $CDCl_3$) δ 7.79 (d, *J* = 8.3 Hz, 2H), 7.28 (d, *J* = 8.1 Hz, 2H), 7.17 (dd, *J* = 7.7, 1.3 Hz, 1H), 7.14-7.11 (m, 1H), 7.06 (d, *J* = 3.4 Hz, 1H), 6.98-6.94 (m, 2H), 6.08 (d, *J* = 3.4 Hz, 1H), 4.43 (dd, *J* = 9.3, 2.6 Hz, 1H), 3.35 (dd, *J* = 16.3, 2.6 Hz, 1H), 3.09 (dd, *J* = 16.3, 9.3 Hz, 1H), 2.48 (s, 3H), 2.39 (s, 3H), 2.34 (s, 3H); ^{13}C NMR (100 MHz, $CDCl_3$) δ 185.4, 161.0, 157.8, 151.3, 150.3, 144.0, 130.0, 129.8, 128.8, 127.9, 127.1, 124.69, 124.3, 119.5, 116.0, 115.3, 108.9, 47.5, 32.0, 21.5, 18.7, 14.0; HRMS (ESI) calcd for $C_{24}H_{22}O_5SNa$ (M+Na) 445.1086; found 445.1060.

2-[6-Methoxy-2-methyl-3-(phenylsulfonyl)-4*H*-chromen-4-yl]-1-phenylethanone (**8fa**). Compound **8fa** (63 mg, 58%) was obtained as a white solid; R_f = 0.6 (30% ethyl acetate in hexane); Mp. 133-135 °C (CH_2Cl_2-hexane); IR ν_{max} (KBr): 3448, 3061, 3004, 2931, 2834, 1682, 1637, 1592, 1493, 1465, 1393, 1355, 1282, 1130, 1091, 1037, 977 cm^{-1}; ^1H NMR (400 MHz, $CDCl_3$) δ 7.92-7.89 (m, 4H), 7.56-7.40 (m, 6H), 6.89 (d, *J* = 8.8 Hz, 1H), 6.72 (d, *J* = 2.8 Hz, 1H), 6.66 (dd, *J* = 8.8, 2.8 Hz, 1H), 4.50 (d, *J* = 9.1 Hz, 1H), 3.67 (s, 3H), 3.59 (dd, *J* = 17.1, 2.3 Hz, 1H), 3.33 (dd, *J* = 17.1, 9.1 Hz, 1H), 2.50 (s, 3H); ^{13}C NMR (125 MHz, $CDCl_3$) δ 197.5, 161.6, 156.4, 144.2, 142.0, 136.8, 133.1(2), 129.2, 128.5, 128.1, 127.0, 125.3, 116.9, 114.2, 114.0, 112.5, 55.6, 48.5, 32.1, 18.8. HRMS (ESI) calcd for $C_{25}H_{22}O_5SNa$ (M+Na) 457.1086; found 457.1074.

2-(6-Methoxy-2-methyl-3-tosyl-4*H*-chromen-4-yl)-1-phenylethanone (**8fb**). Compound **8fb** (68 mg, 61%) was obtained as a white solid; R_f = 0.6 (30% ethyl acetate in hexane); Mp. 119-121 °C (CH_2Cl_2-hexane); IR ν_{max} (KBr): 3448, 2925, 1676, 1637, 1495, 1448, 1437, 1395, 1331, 1212, 1148, 1091 cm^{-1}; ^1H NMR (500 MHz, $CDCl_3$) δ 7.92-7.84 (m, 2H), 7.78 (d, *J* = 8.4 Hz, 2H),

7.54-7.51 (m, 1H), 7.43-7.40 (m, 2H), 7.27 (d, J = 8.4 Hz, 2H), 6.88 (d, J = 8.9 Hz, 1H), 6.72 (d, J = 2.9 Hz, 1H), 6.66 (dd, J = 8.9, 2.5 Hz, 1H), 4.48 (dd, J = 9.1, 2.1 Hz, 1H), 3.67 (s, 1H), 3.57 (dd, J = 17.1, 2.5 Hz, 1H), 3.31 (dd, J = 17.1, 9.1 Hz, 1H), 2.49 (s, 3H), 2.38 (s, 3H); ^{13}C NMR (100 MHz, $CDCl_3$) δ 197.5, 161.2, 156.3, 144.3, 144.0, 139.0, 136.8, 133.1, 129.8, 128.5, 128.1, 127.0, 125.3, 116.8, 114.3, 114.1, 112.5, 55.6, 48.5, 32.1, 21.5, 18.7; HRMS (ESI) calcd for $C_{26}H_{24}O_5SNa$ (M+Na) 471.1242; found 471.1215.

2-[6-Chloro-2-methyl-3-(phenylsulfonyl)-4*H*-chromen-4-yl]-1-phenylethanone (**8ga**). Compound **8ga** (77 mg, 64%) was obtained as a white solid; R_f = 0.7 (30% ethyl acetate in hexane); Mp. 116-118 °C (CH_2Cl_2-hexane); IR ν_{max} (KBr): 3448, 2924, 1682, 1654, 1635, 1586, 1488, 1461, 1282, 1205, 1090, 978, 815, 771 cm^{-1}; ^{1}H NMR (500 MHz, $CDCl_3$) δ 7.90-7.89 (m, 4H), 7.57-7.49 (m, 4H), 7.44-7.41 (m, 2H), 7.24 (d, J = 1.6 Hz, 1H), 7.09 (dd, J = 8.6, 1.6 Hz, 1H), 6.90 (d, J = 8.6 Hz, 1H), 4.47 (d, J = 8.8 Hz, 1H), 3.57 (d, J = 17.4 Hz, 1H), 3.35 (dd, J = 17.4, 8.8 Hz, 1H), 2.51 (s, 3H); ^{13}C NMR (75 MHz, $CDCl_3$) δ 196.9, 161.3, 148.9, 141.6, 136.6, 133.3, 129.7, 129.3, 129.0, 128.9, 128.6, 128.4, 128.1, 127.0, 126.2, 117.4, 114.9, 48.1, 31.5, 18.6; HRMS (ESI) calcd for $C_{24}H_{19}ClO_4SNa$ (M+Na) 461.0590; found 461.0570.

2-(6-Chloro-2-methyl-3-tosyl-4*H*-chromen-4-yl)-1-phenylethanone (**8gb**). Compound **8gb** (75 mg, 66%) was obtained as a white solid; R_f = 0.7 (30% ethyl acetate in hexane); Mp. 137-139 °C (CH_2Cl_2-hexane); IR ν_{max} (KBr): 3442, 3089, 2923, 2581, 1685, 1639, 1448, 1419, 1396, 1293, 1278, 1149, 1050, 1000, 820, 755 cm^{-1}; ^{1}H NMR (500 MHz, $CDCl_3$) δ 7.88 (d, J = 7.5 Hz, 2H), 7.77 (d, J = 8.1 Hz, 2H), 7.55-7.52 (m, 1H), 7.44-7.41 (m, 2H), 7.28 (d, J = 8.1 Hz, 2H), 7.23 (d, J = 2.2 Hz, 1H), 7.08 (dd, J = 8.6, 2.2 Hz, 1H), 6.89 (d, J = 8.6 Hz, 1H), 4.45 (d, J = 8.8 Hz, 1H), 3.56 (d, J = 17.5 Hz, 1H), 3.33 (dd, J = 17.5, 8.8 Hz, 1H), 2.51 (s, 3H), 2.38 (s, 2H); ^{13}C NMR (100 MHz, $CDCl_3$) δ 197.0, 160.9, 148.9, 144.3, 138.7, 136.6, 133.2, 129.9, 129.6, 128.6, 128.4, 128.1, 128.0, 127.1, 126.3, 117.4, 115.1, 48.0, 31.4, 21.6, 18.6; HRMS (ESI) calcd for $C_{25}H_{21}ClO_4SNa$ (M+Na) 475.0747; found 475.0728.

2-[6-Bromo-2-methyl-3-(phenylsulfonyl)-4*H*-chromen-4-yl]-1-phenylethanone (**8ha**). Compound **8ha** (72 mg, 60%) was obtained as a white solid; R_f = 0.6 (30% ethyl acetate in hexane); Mp. 163-165 °C (CH_2Cl_2-hexane); IR ν_{max} (KBr): 3448, 2921, 2361, 1682, 1641, 1597, 1575, 1479, 1447, 1414, 1396, 1208, 1191, 1072, 990, 975 cm^{-1}; ^{1}H NMR (500 MHz, $CDCl_3$) δ 7.90-7.88 (m, 4H), 7.59-7.49 (m, 4H), 7.45-7.42 (m, 2H), 7.38 (d, J = 2.3 Hz, 1H), 7.24 (dd, J = 8.7, 2.3 Hz, 1H), 4.46 (dd, J = 8.7, 2.2 Hz, 1H), 3.57 (dd, J = 17.5, 2.2 Hz, 1H), 3.35 (dd, J = 17.5, 8.7 Hz, 1H), 2.51 (s, 3H), 1.56 (s, 3H); ^{13}C NMR (125 MHz, $CDCl_3$) δ 196.9, 161.2, 149.4, 141.57, 136.5, 133.3, 133.3, 131.3, 131.0, 129.3, 128.6, 128.1, 127.0, 126.6, 117.8, 117.1, 114.9, 48.0, 31.4, 18.6; HRMS (ESI) calcd for $C_{24}H_{19}BrO_4SNa$ (M+Na) 505.0085; found 505.0069.

2-(6-Bromo-2-methyl-3-tosyl-4*H*-chromen-4-yl)-1-phenylethanone (**8hb**). Compound **8hb** (76 mg, 61%) was obtained as a white solid; R_f = 0.6 (30% ethyl acetate in hexane); Mp. 181-183 °C (CH_2Cl_2-hexane); IR ν_{max} (KBr): 3442, 2923, 1653, 1637, 1596, 1578, 1477, 1448, 1313, 1205, 1182, 1148, 1089, 814, 682 cm^{-1}; ^{1}H NMR (400 MHz, $CDCl_3$) δ 7.88 (d, J = 7.2 Hz, 1H), 7.76

(d, *J* = 7.0 Hz, 1H), 7.54-7.52 (m, 1H), 7.45-7.41 (m, 2H), 7.37 (s, 1H), 7.29-7.22 (m, 3H), 6.84 (d, *J* = 8.4 Hz, 1H), 4.44 (d, *J* = 8.8 Hz, 1H), 3.56 (d, *J* = 17.5 Hz, 1H), 3.34 (dd, *J* = 17.5, 8.8 Hz, 1H), 2.51 (s, 3H), 2.38 (s, 3H); ^{13}C NMR (100 MHz, $CDCl_3$) δ 196.9, 160.8, 149.41, 144.3, 138.6, 136.5, 133.2, 131.3, 130.9, 129.9, 128.5, 128.1, 127.1, 126.7, 117.8, 117.0, 115.2, 48.0, 31.3, 21.5, 18.6; HRMS (ESI) calcd for $C_{25}H_{21}BrO_4SNa$ (M+Na) 519.0242; found 519.0222.

2-[6,8-Dichloro-2-methyl-3-(phenylsulfonyl)-4*H*-chromen-4-yl]-1-phenylethanone (**8ia**). Compound **8ia** (72 mg, 61%) was obtained as a white solid; R_f = 0.6 (30% ethyl acetate in hexane); Mp. 136-138 $^{\circ}$C (CH_2Cl_2-hexane); IR ν_{max} (KBr): 3435, 3064, 2924, 2360, 1676, 1643, 1597, 1549, 1459, 1320, 1215, 1176, 1153, 1089, 755 cm^{-1}; ^{1}H NMR (400 MHz, $CDCl_3$) δ 7.91-7.87 (m, 4H), 7.59-7.50 (m, 4H), 7.44 (t, *J* = 7.7 Hz, 2H), 7.20-7.18 (m, 2H), 4.49 (dd, *J* = 8.9, 2.1 Hz, 1H), 3.58 (dd, *J* = 17.7, 2.1 Hz, 1H), 3.35 (dd, *J* = 17.7, 8.9 Hz, 1H), 2.58 (s, 3H); ^{13}C NMR (100 MHz, $CDCl_3$, 1 signal is not observed in spectrum possibly due to overlapping signals) δ 196.6, 161.1, 145.1, 141.2, 136.3, 133.5, 133.4, 129.5, 129.4, 128.6, 128.50, 128.1, 127.5, 127.1, 122.3, 115.8, 47.9, 31.7, 18.5; HRMS (ESI) calcd for $C_{24}H_{18}Cl_2O_4SNa$ (M+Na) 495.0201; found 495.0185.

2-(6,8-Dichloro-2-methyl-3-tosyl-4*H*-chromen-4-yl)-1-phenylethanone (**8ib**). Compound **8ib** (81 mg, 67%) was obtained as a white solid; R_f = 0.6 (30% ethyl acetate in hexane); Mp. 141-143 $^{\circ}$C (CH_2Cl_2-hexane); IR ν_{max} (KBr): 3434, 30613, 2922, 2359, 2342, 1686, 1641, 1597, 1460, 1448, 1423, 1332, 1312, 1291, 1273, 1185, 1149, 1092, 755 cm^{-1}; ^{1}H NMR (500 MHz, $CDCl_3$) δ 7.88 (d, *J* = 7.4 Hz, 2H), 7.77 (d, *J* = 8.1 Hz, 2H), 7.57-7.54 (m, 1H), 7.45-7.42 (m, 2H), 7.30 (d, *J* = 8.1 Hz, 1H), 7.19 (d, *J* = 2.3 Hz, 1H), 7.18 (d, *J* = 2.3 Hz, 1H), 4.48-4.46 (m, 1H), 3.56 (dd, *J* = 17.7, 2.2 Hz, 1H), 3.33 (dd, *J* = 17.7, 9.1 Hz, 1H), 2.58 (s, 3H), 2.39 (s, 3H); ^{13}C NMR (100 MHz, $CDCl_3$) δ 196.6, 160.7, 145.2, 144.5, 138.3, 136.3, 133.4, 130.0, 129.4, 128.6, 128.4, 128.1, 127.6, 127.1, 127.0, 122.2, 116.0, 47.8, 31.6, 21.6, 18.4; HRMS (ESI) calcd for $C_{25}H_{20}Cl_2O_4SNa$ (M+Na) 509.0357; found 509.0340.

^{1}H NMR spectrum of **7d** (300 MHz, $CDCl_3$+DMSO)

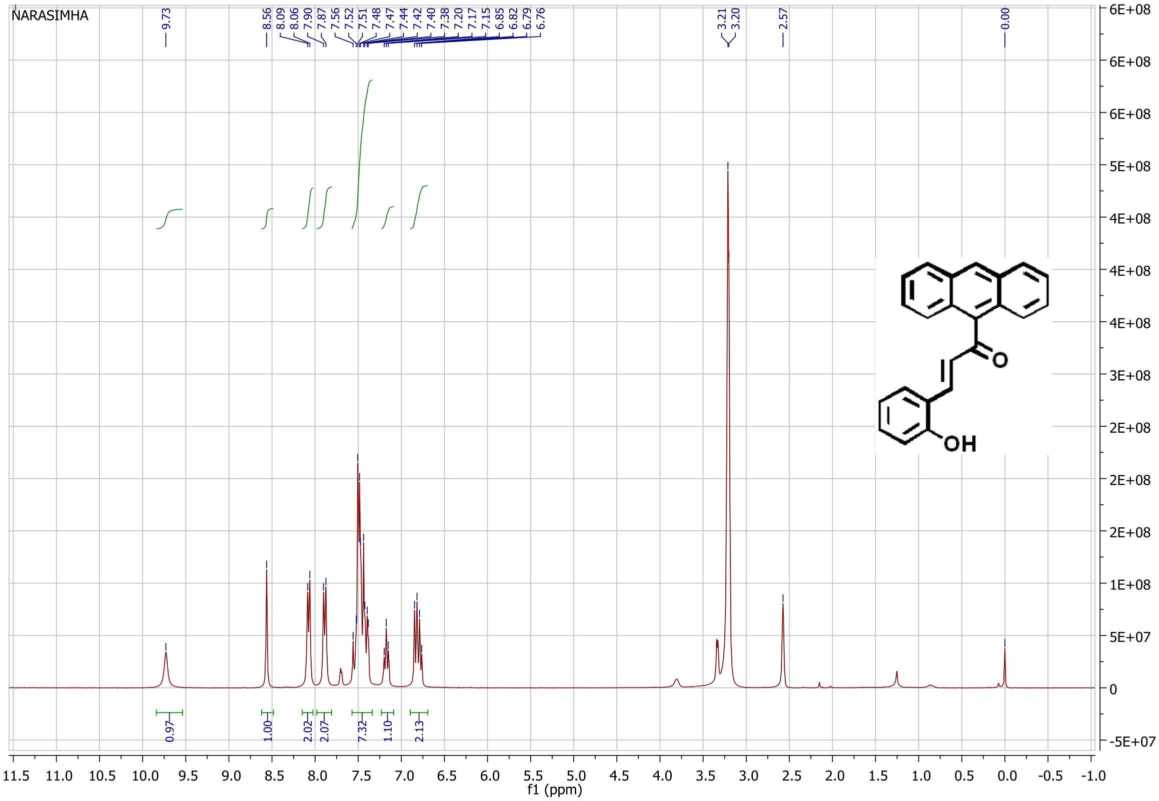

^{13}C NMR spectrum of **7d** (75 MHz, $CDCl_3$+DMSO)

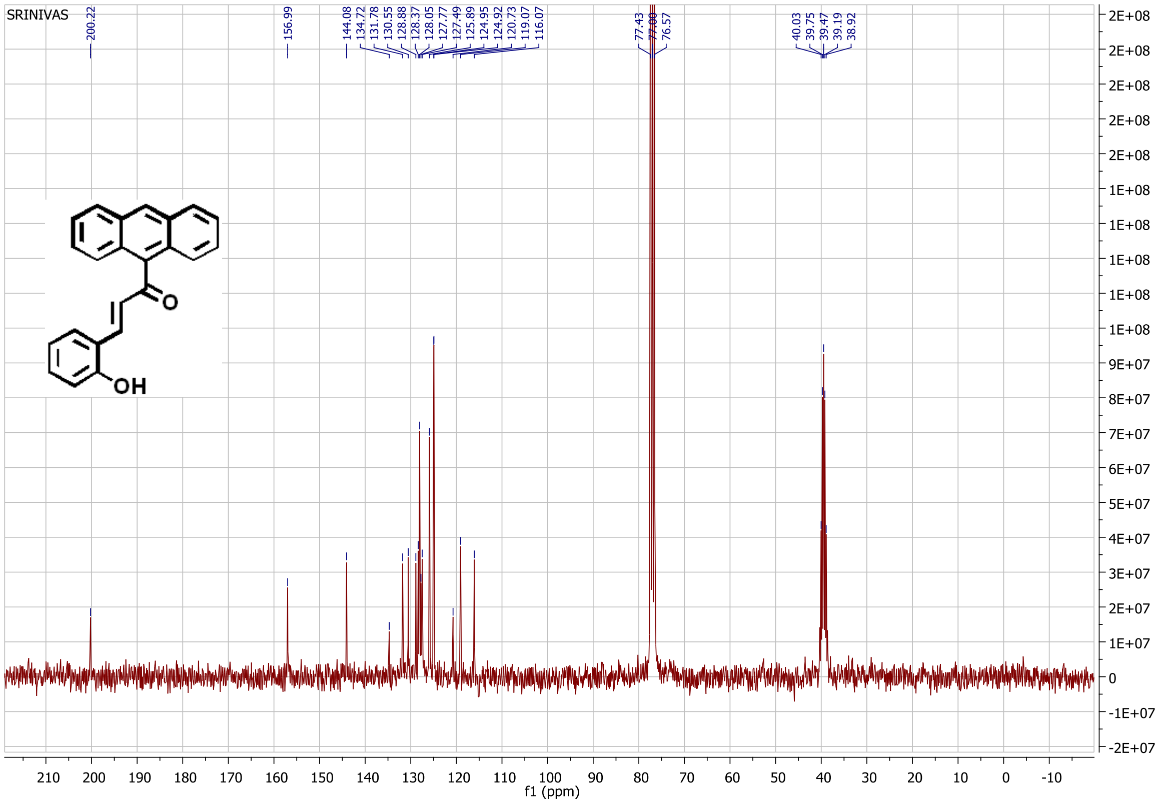

^{1}H NMR spectrum of **8aa** (400 MHz, $CDCl_3$)

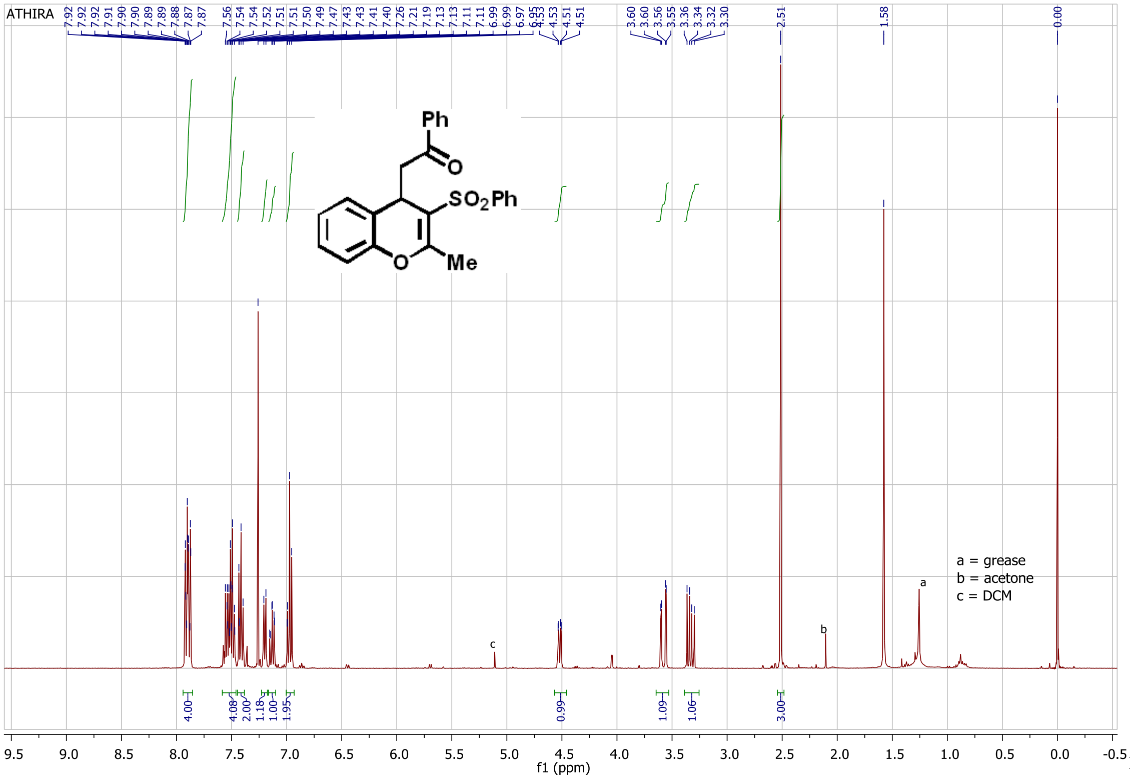

^{13}C NMR spectrum of **8aa** (125 MHz, $CDCl_3$)

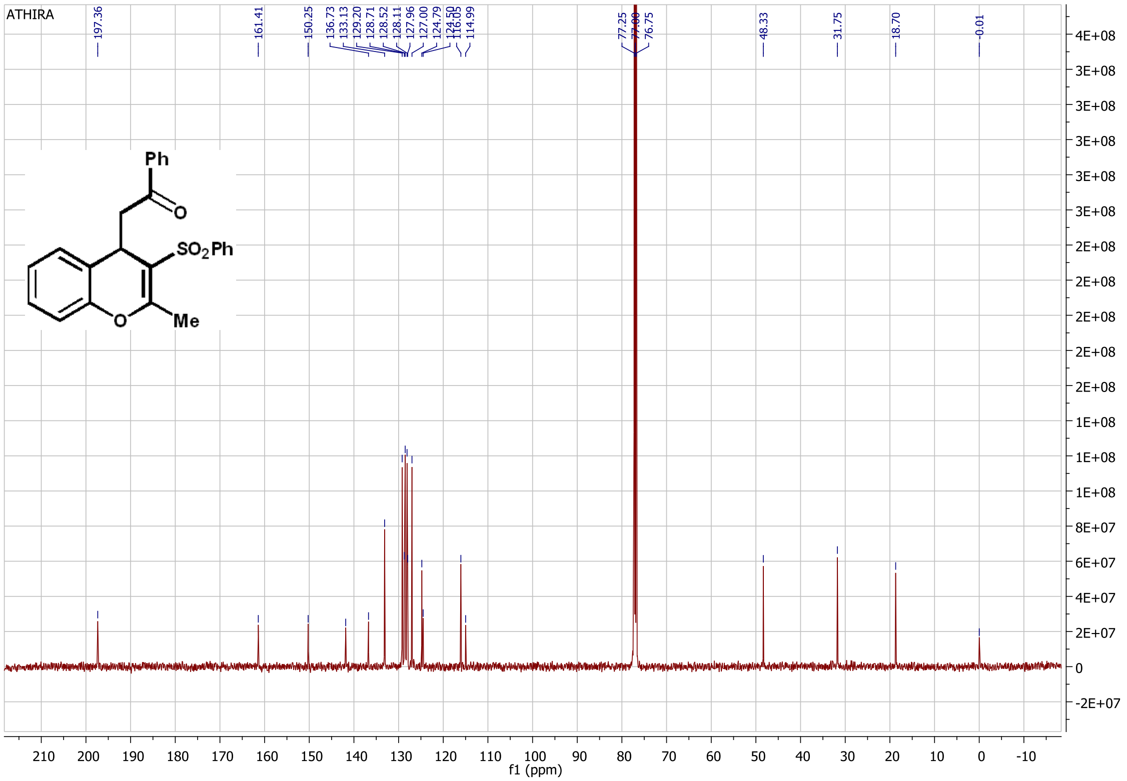

^{1}H NMR spectrum of **8ab** (400 MHz, $CDCl_3$)

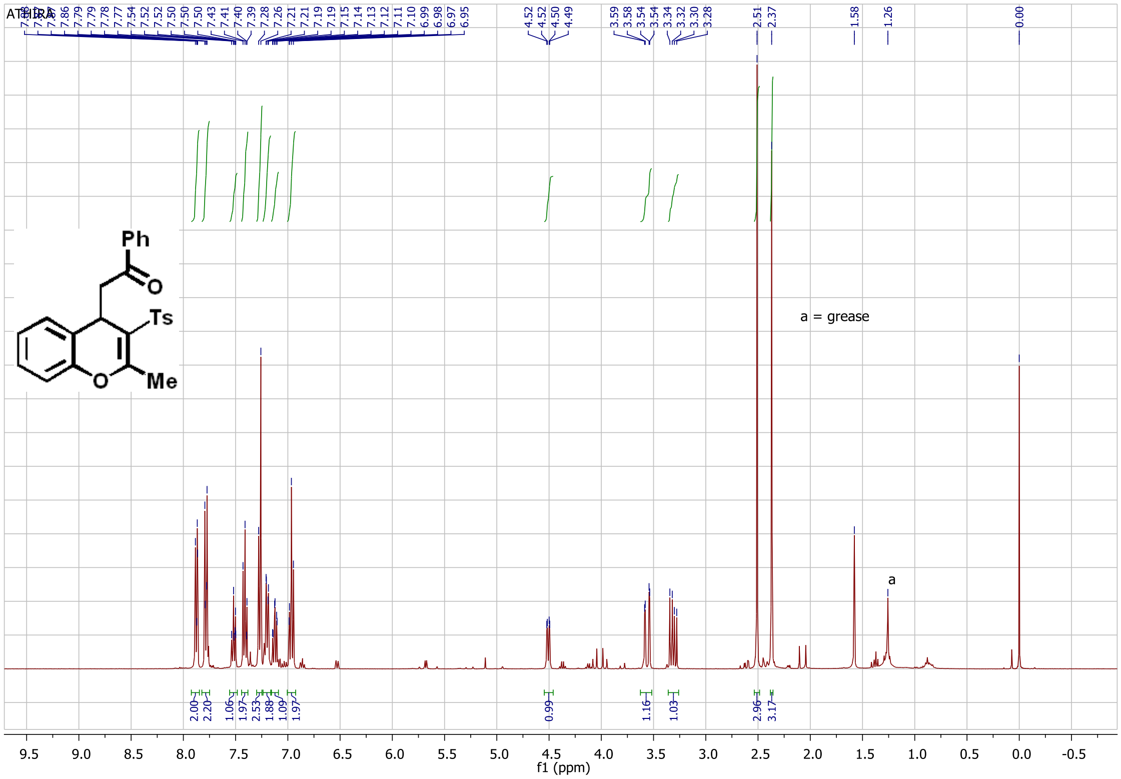

^{13}C NMR spectrum of **8ab** (100 MHz, $CDCl_3$)

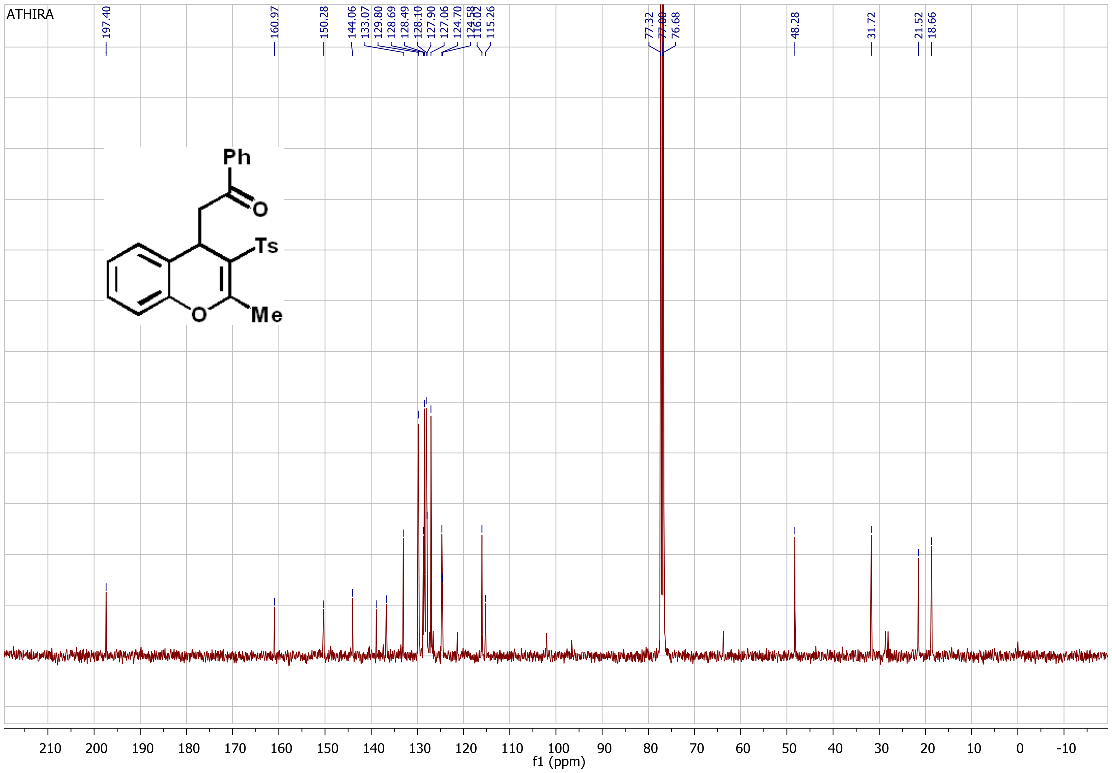

^{1}H NMR spectrum of **8ba** (400 MHz, $CDCl_3$)

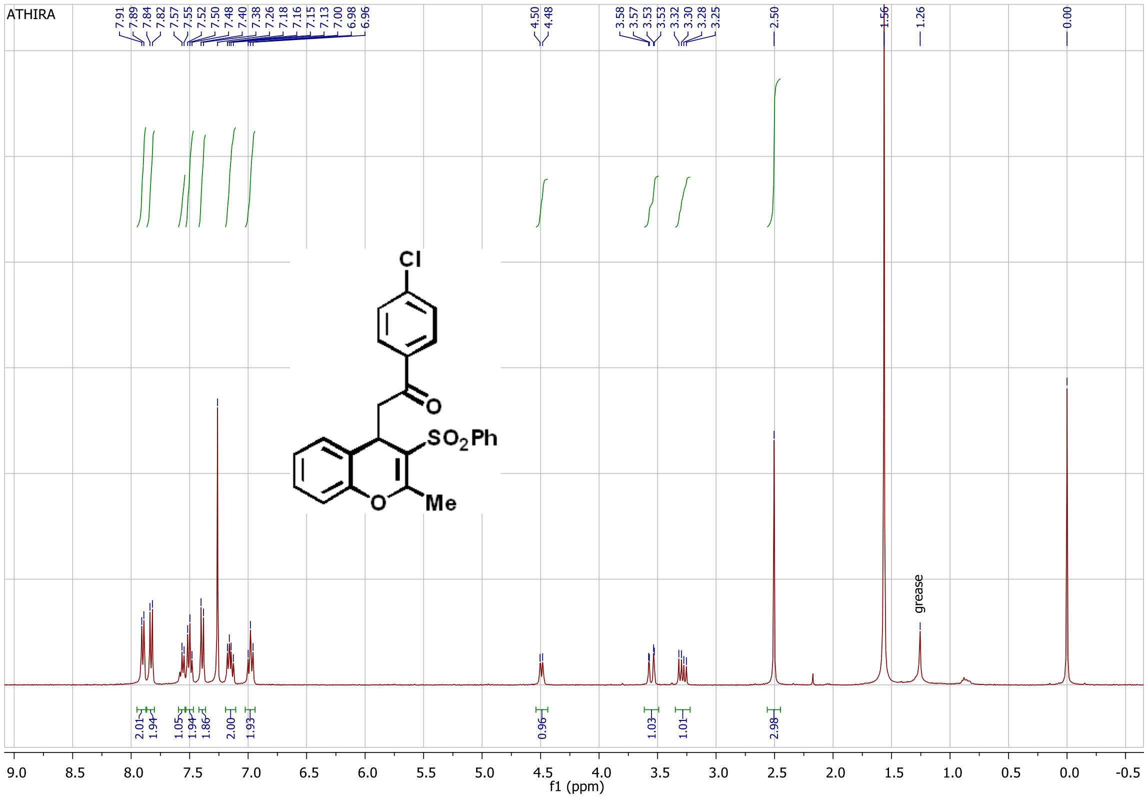

^{13}C NMR spectrum of **8ba** (100 MHz, $CDCl_3$)

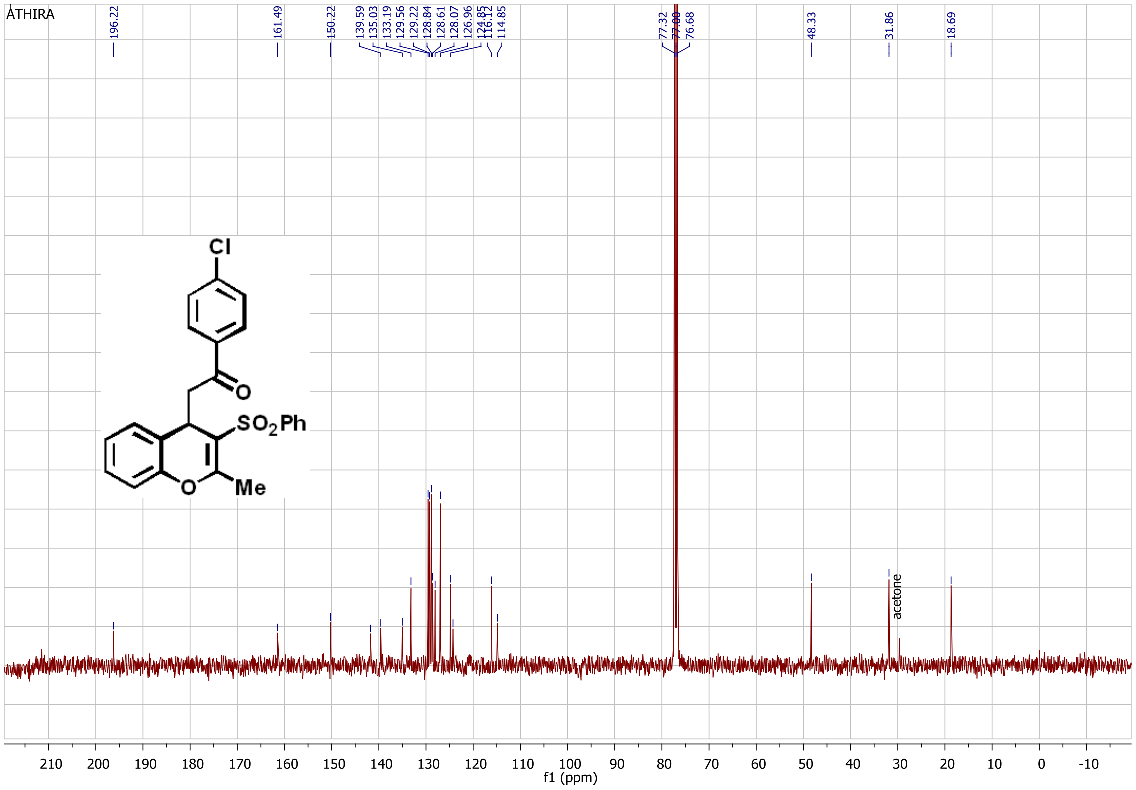

^{1}H NMR spectrum of **8bb** (400 MHz, $CDCl_3$)

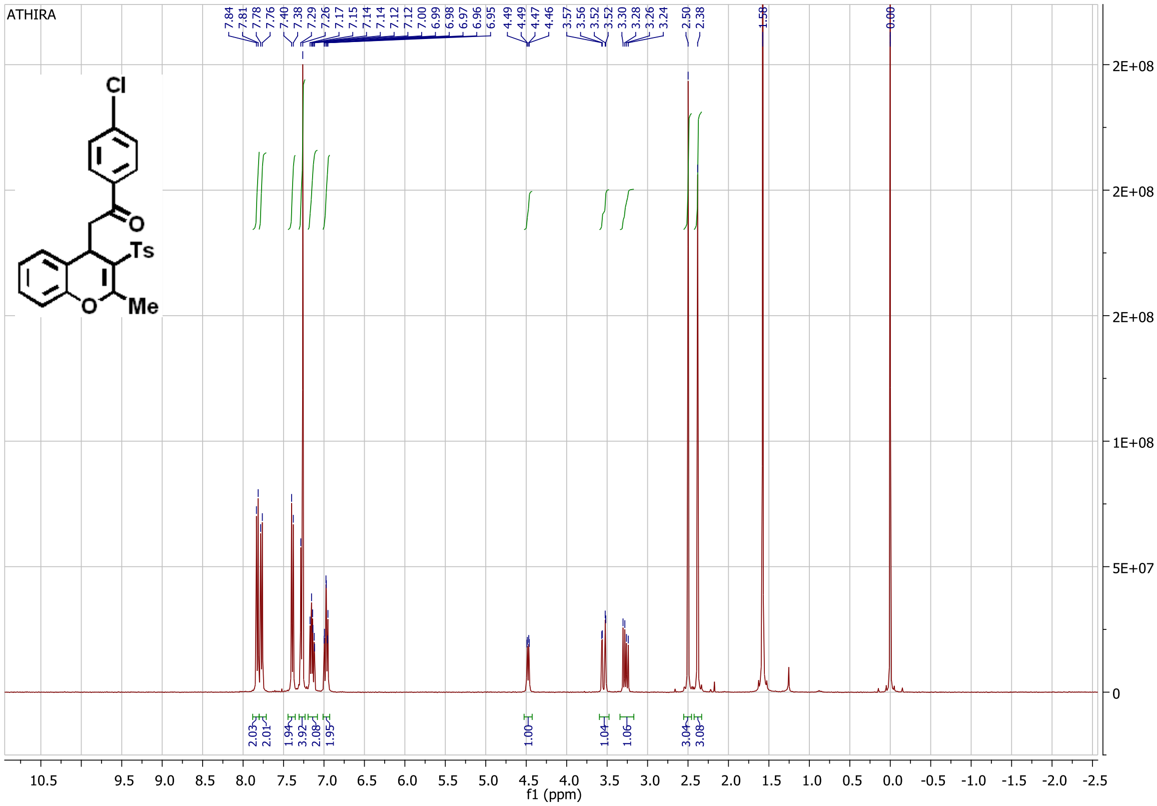

^{13}C NMR spectrum of **8bb** (100 MHz, $CDCl_3$)

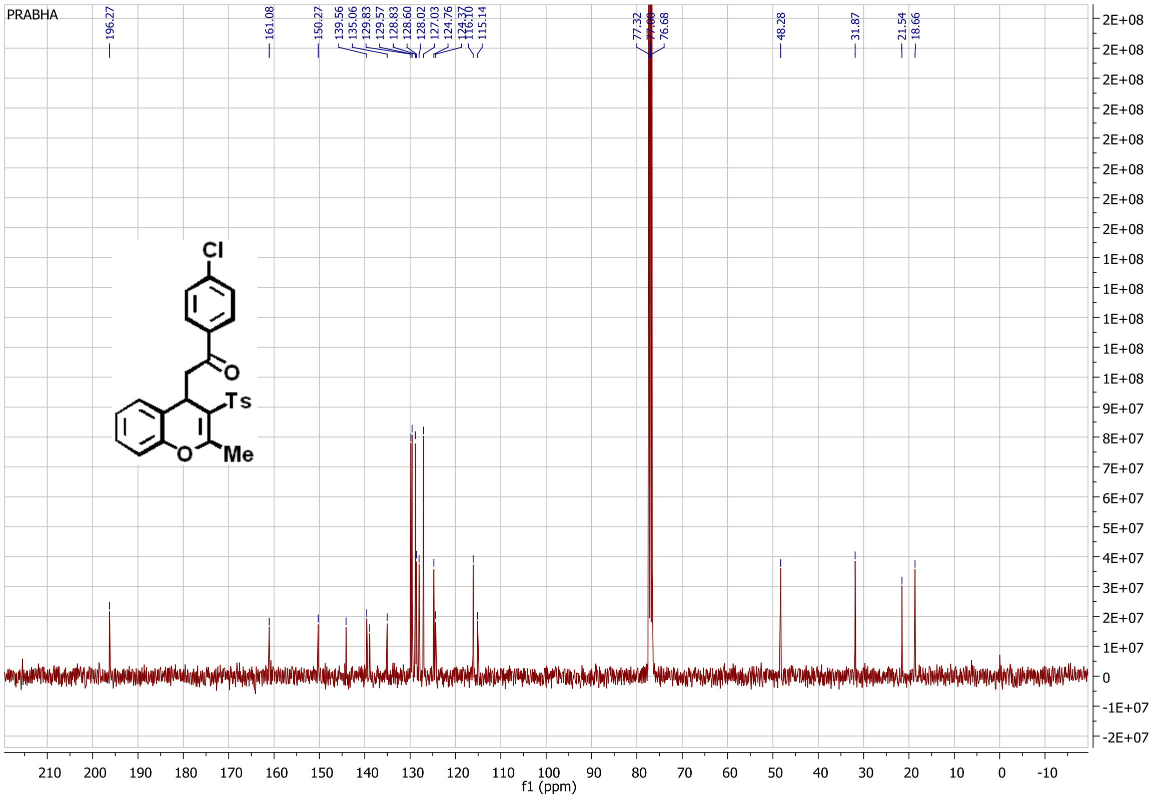

^{1}H NMR spectrum of **8ca** (500 MHz, $CDCl_3$)

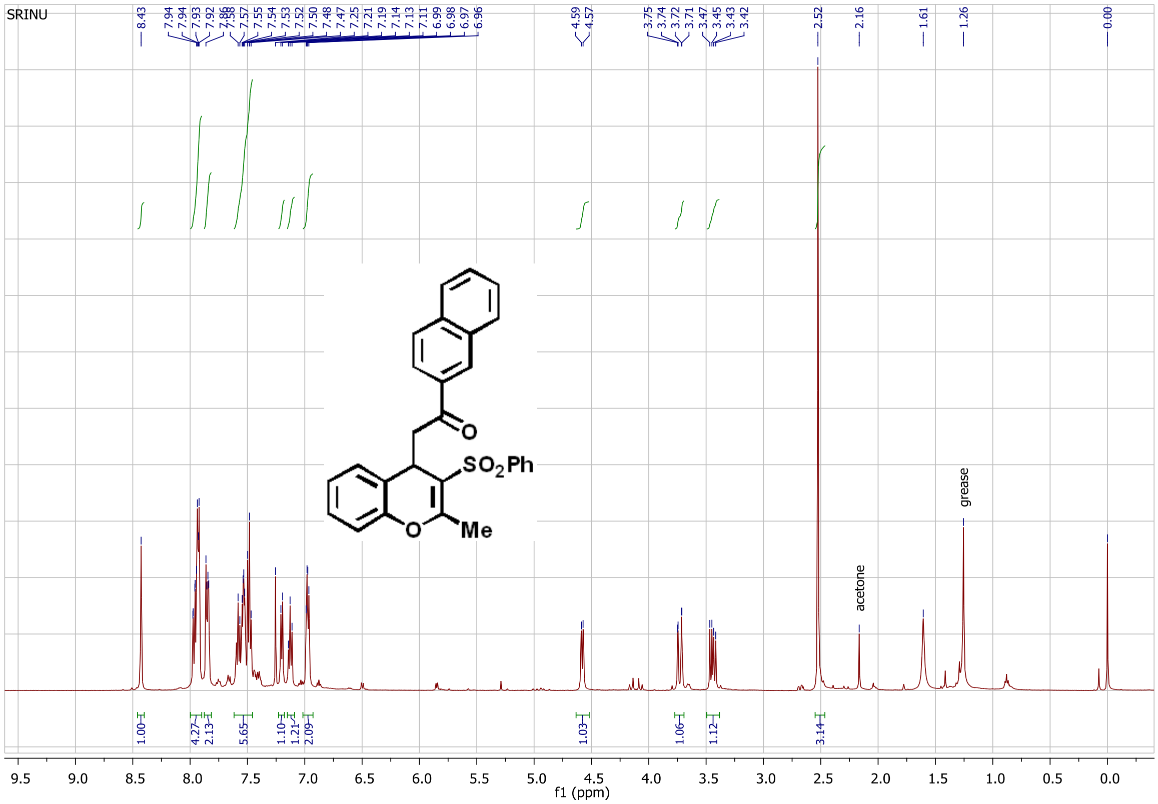

^{13}C NMR spectrum of **8ca** (100 MHz, $CDCl_3$)

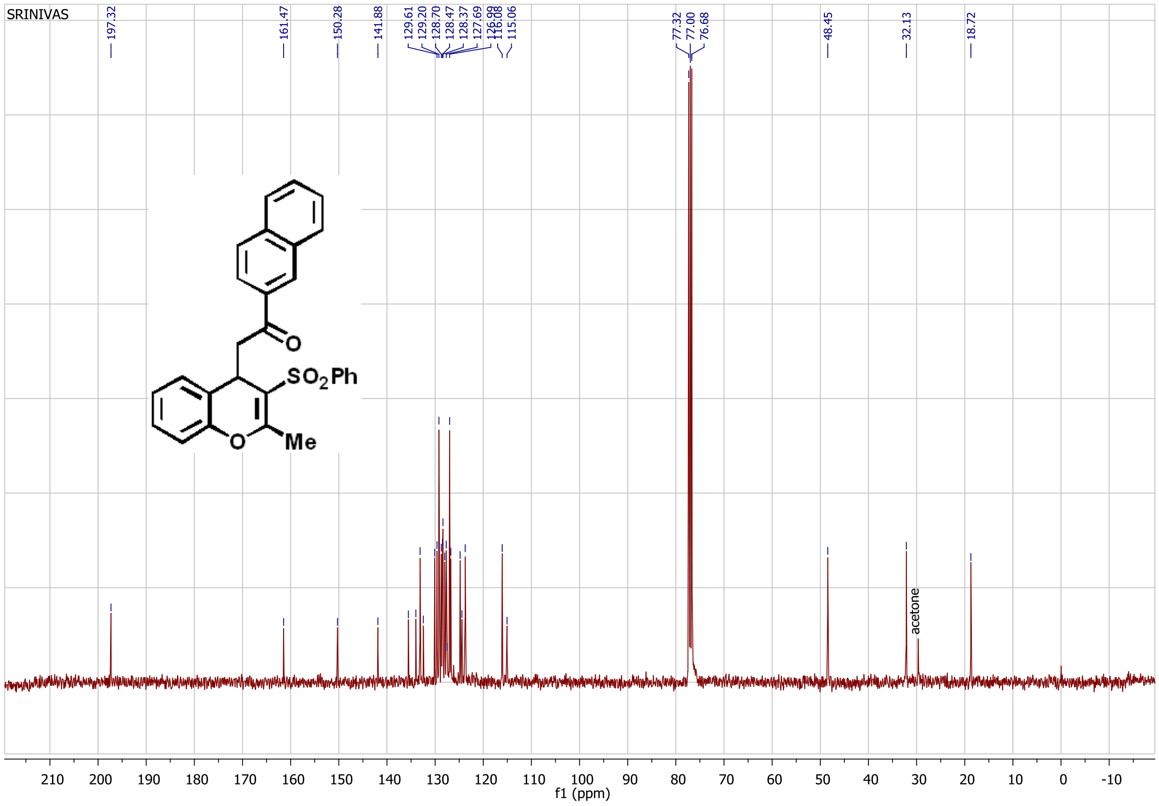

^{1}H NMR spectrum of **8cb** (500 MHz, $CDCl_3$)

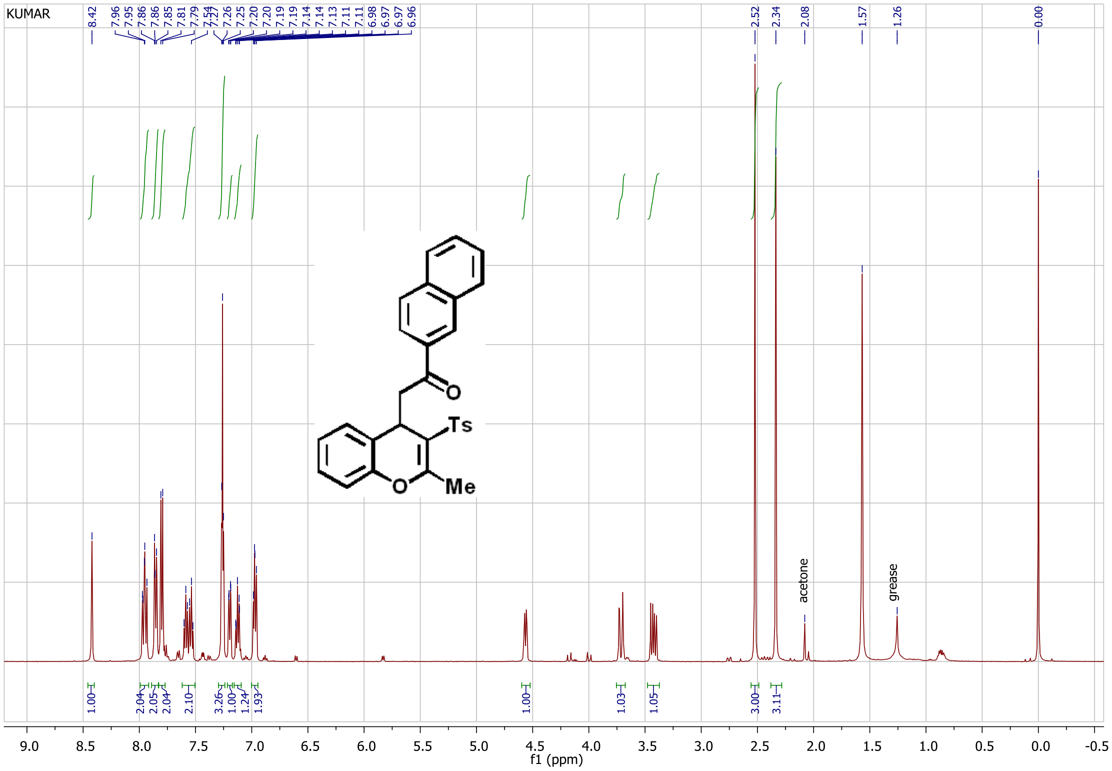

^{13}C NMR spectrum of **8cb** (100 MHz, $CDCl_3$)

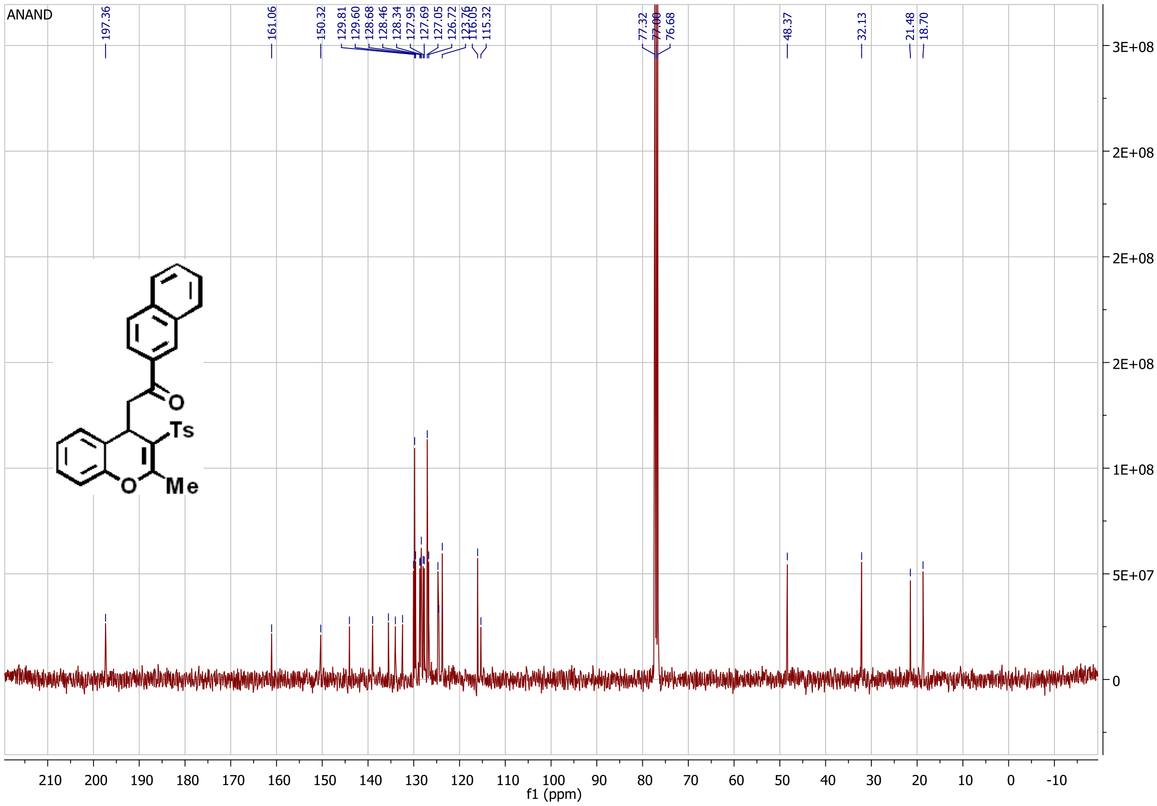

^{1}H NMR spectrum of **8da** (500 MHz, $CDCl_3$)

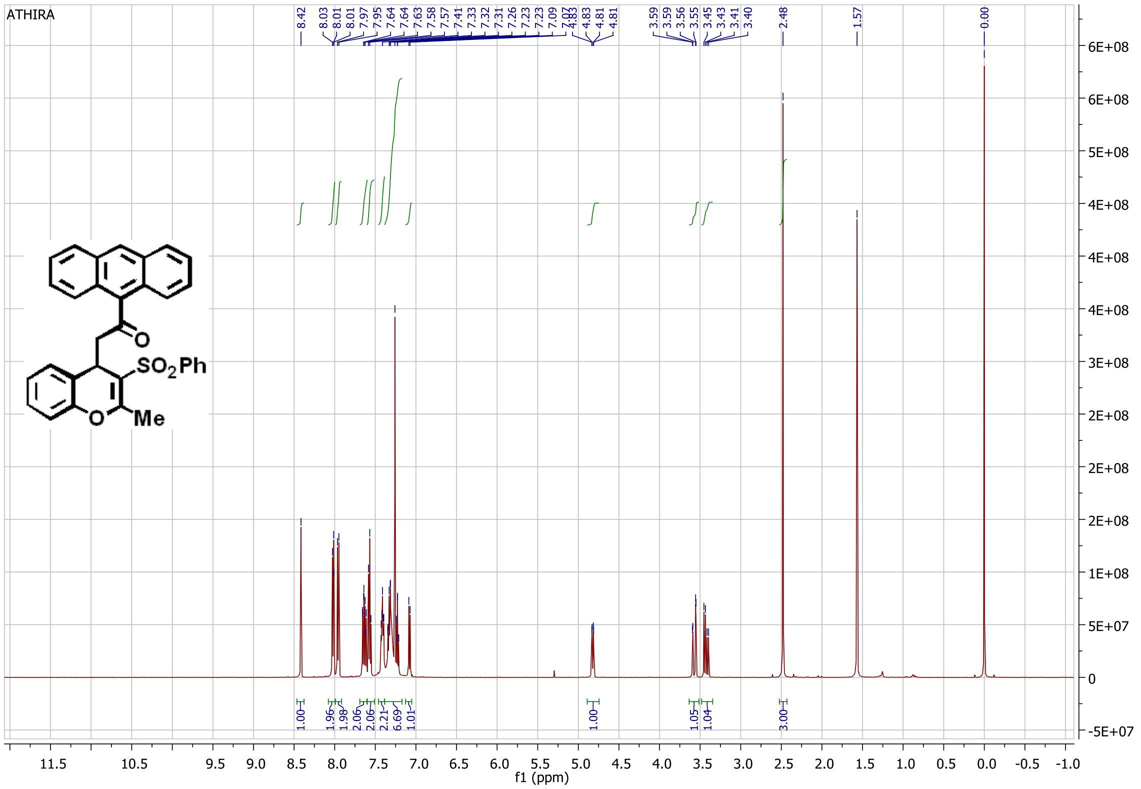

^{13}C NMR spectrum of **8da** (125 MHz, $CDCl_3$)

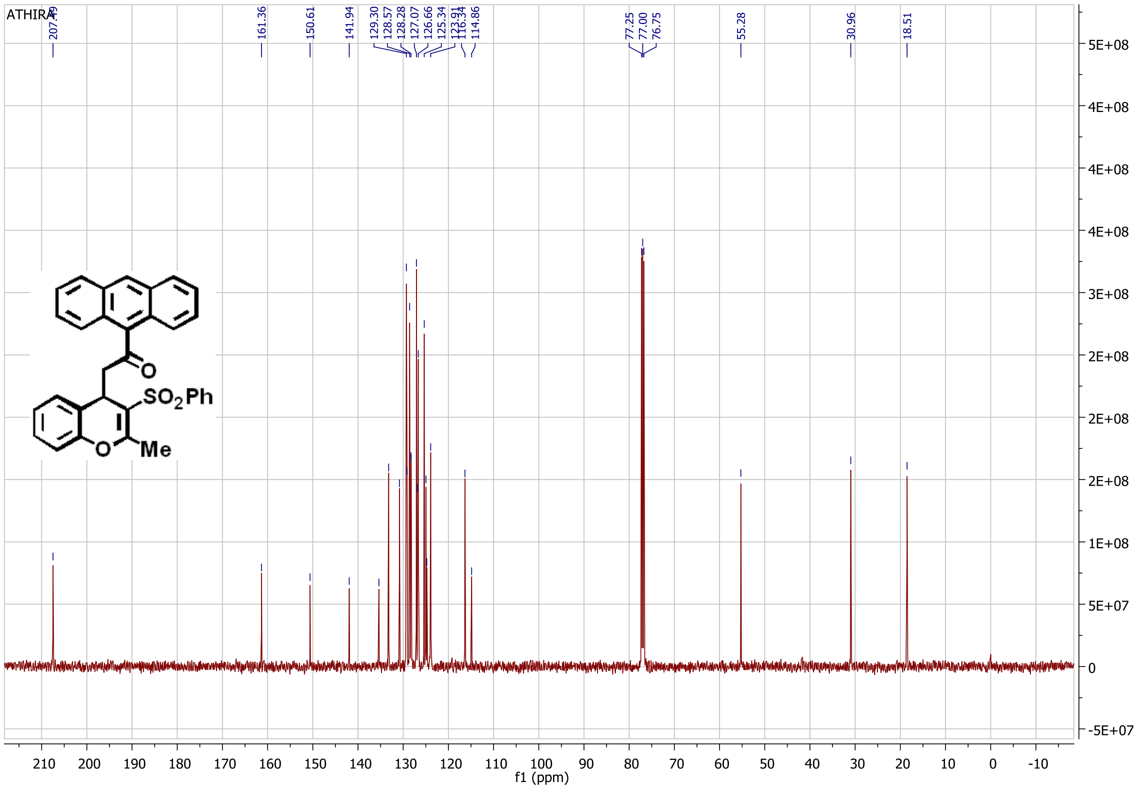

^{1}H NMR spectrum of **8db** (500 MHz, $CDCl_3$)

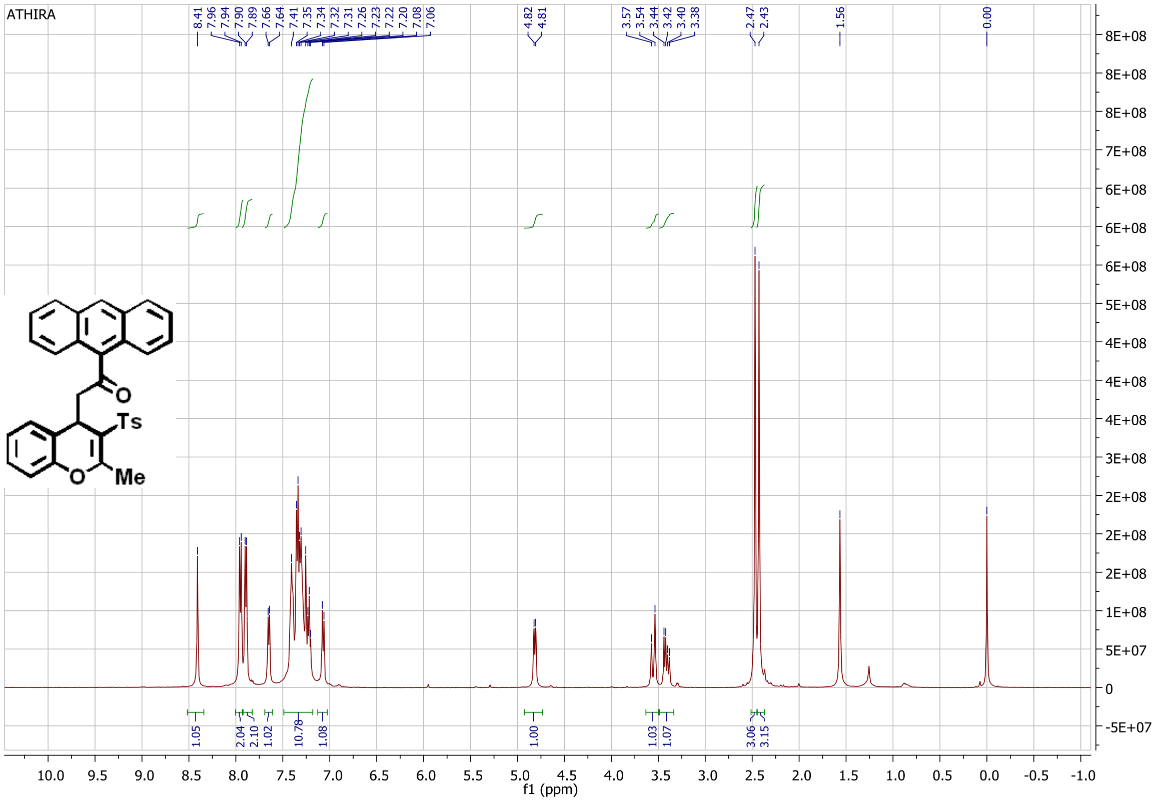

^{13}C NMR spectrum of **8db** (100 MHz, $CDCl_3$)

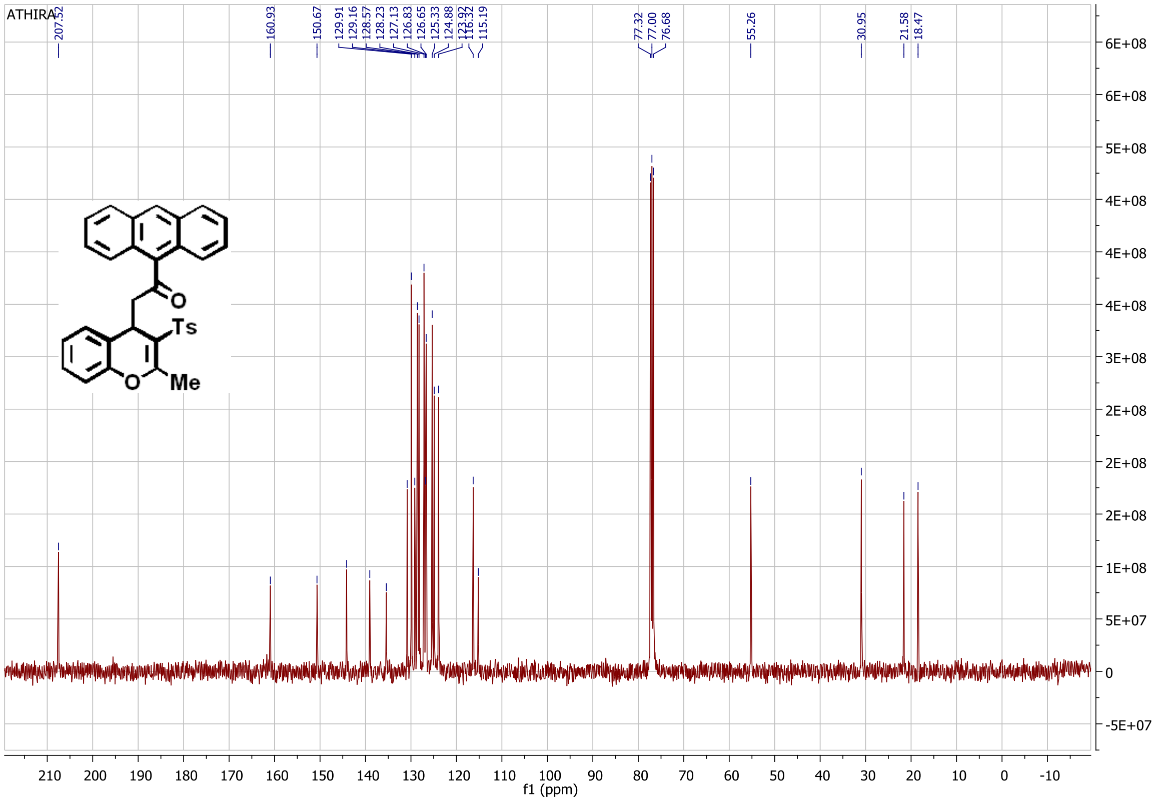

^{1}H NMR spectrum of **8ea** (500 MHz, $CDCl_3$)

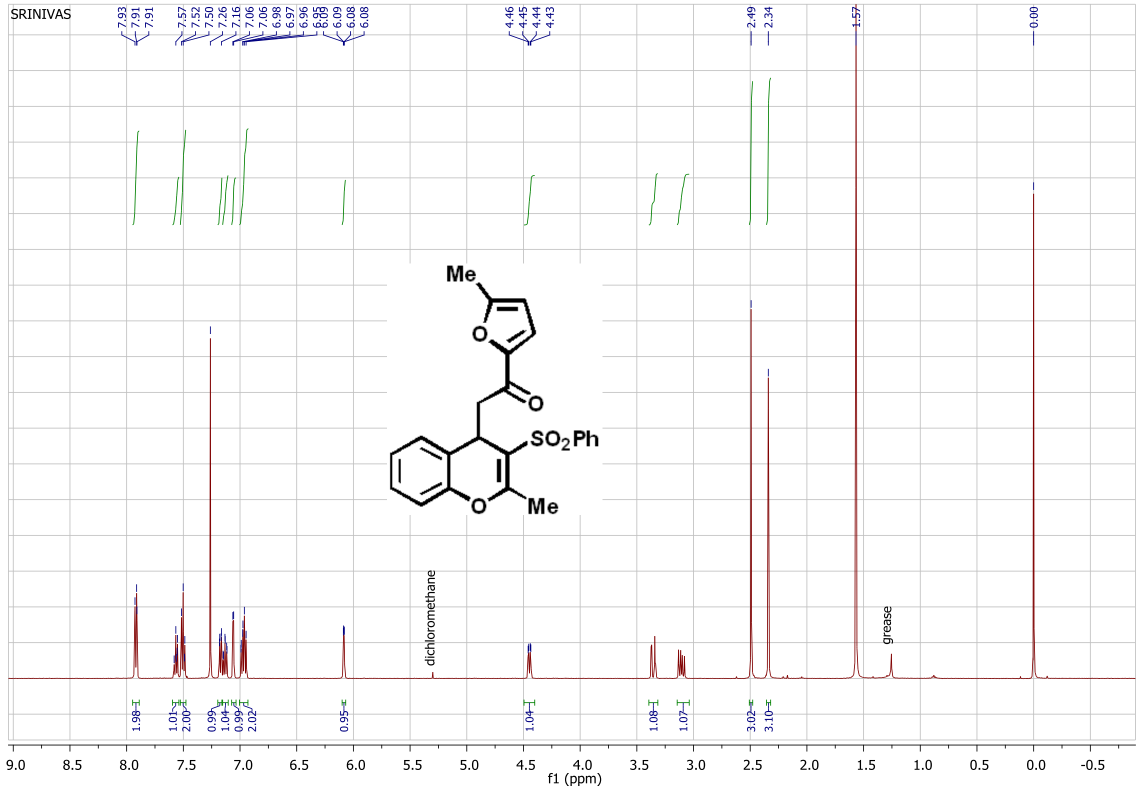

^{13}C NMR spectrum of **8ea** (100 MHz, $CDCl_3$)

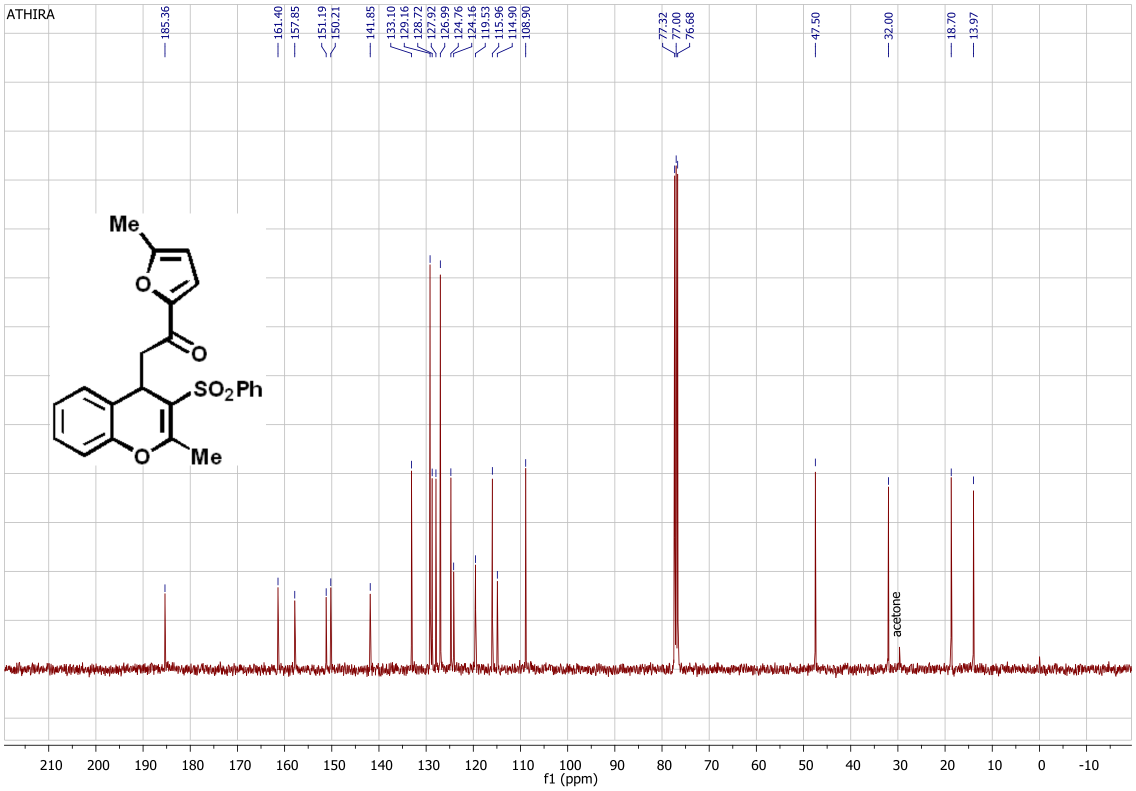

^{1}H NMR spectrum of **8eb** (500 MHz, $CDCl_3$)

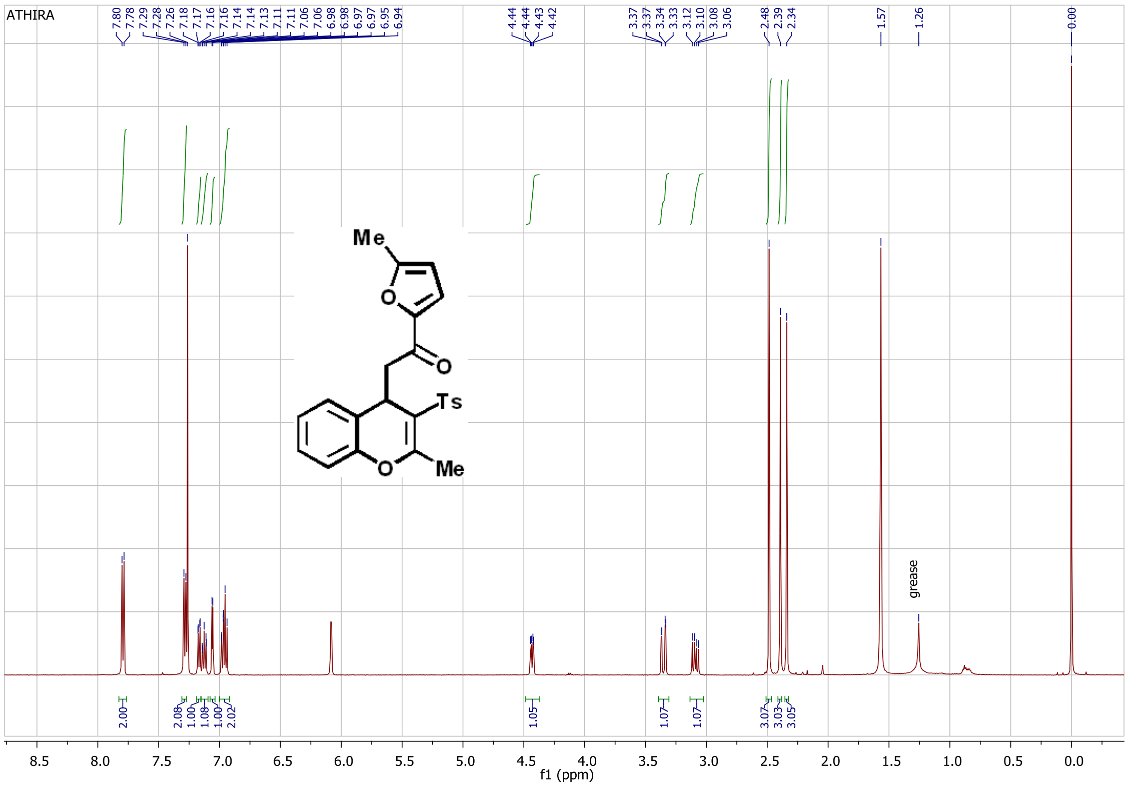

^{13}C NMR spectrum of **8eb** (100 MHz, $CDCl_3$)

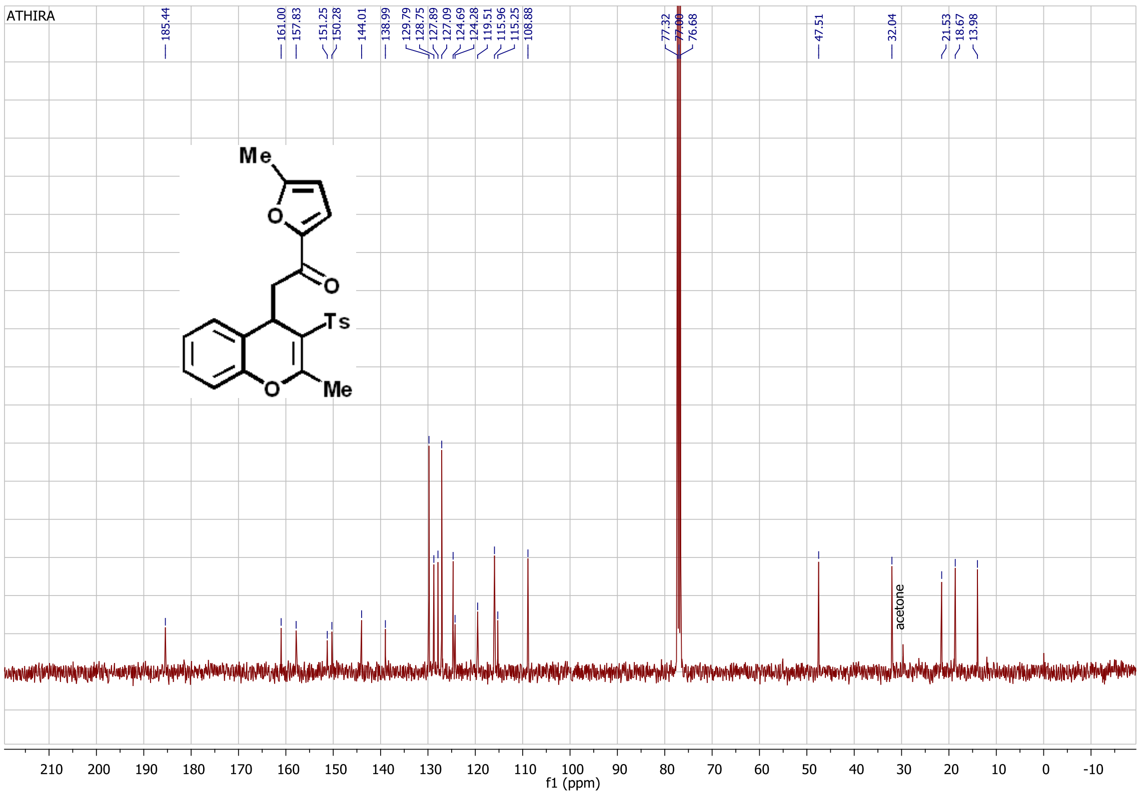

^{1}H NMR spectrum of **8fa** (400 MHz, $CDCl_3$)

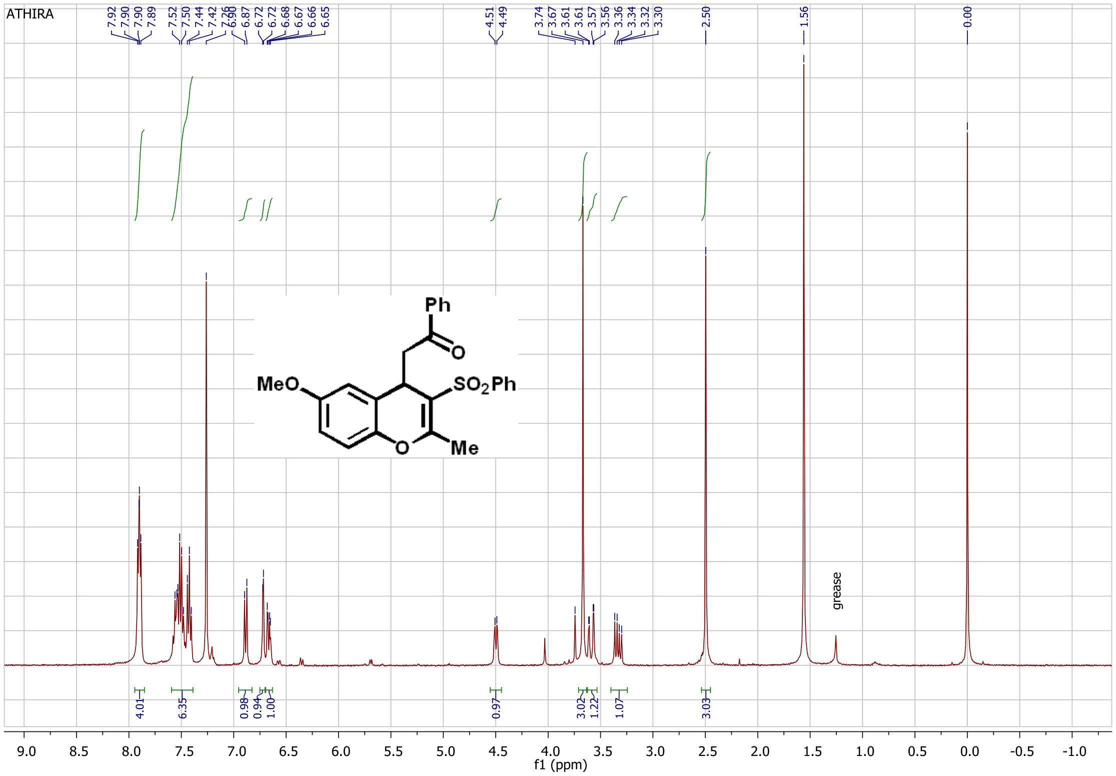

^{13}C NMR spectrum of **8fa** (125 MHz, $CDCl_3$)

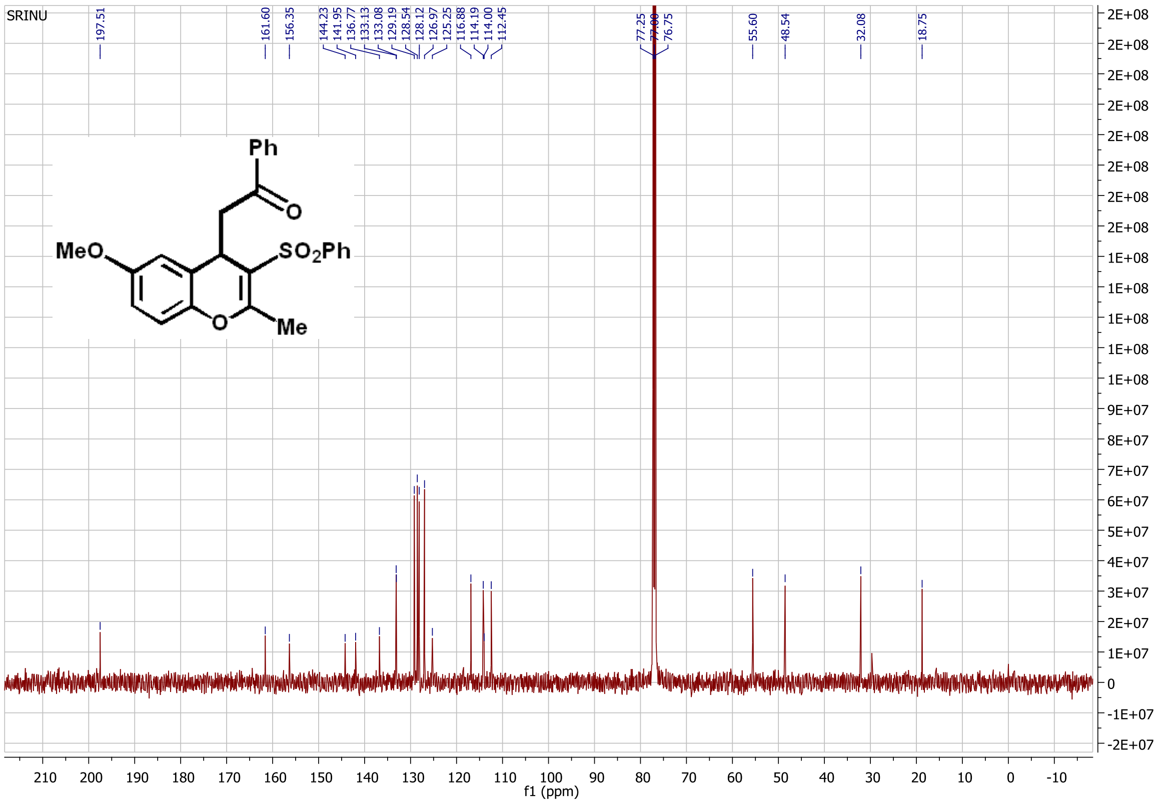

^{1}H NMR spectrum of **8fb** (500 MHz, $CDCl_3$)

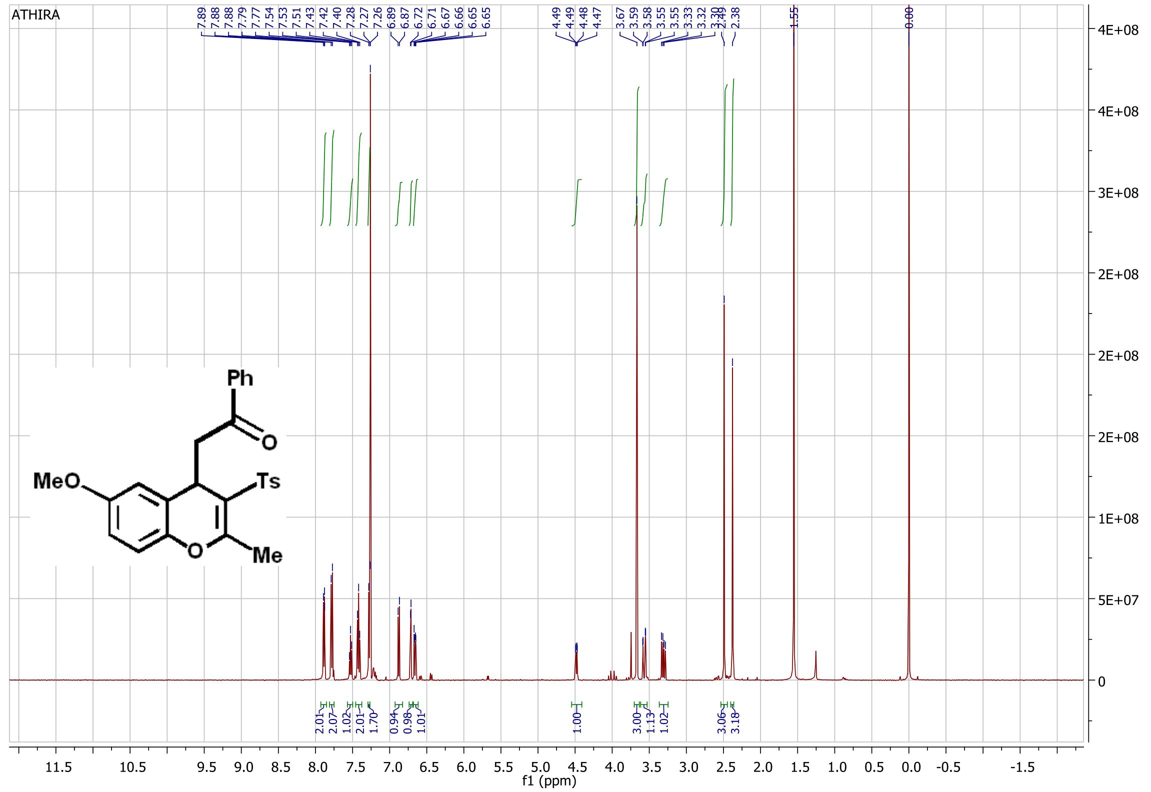

^{13}C NMR spectrum of **8fb** (100 MHz, $CDCl_3$)

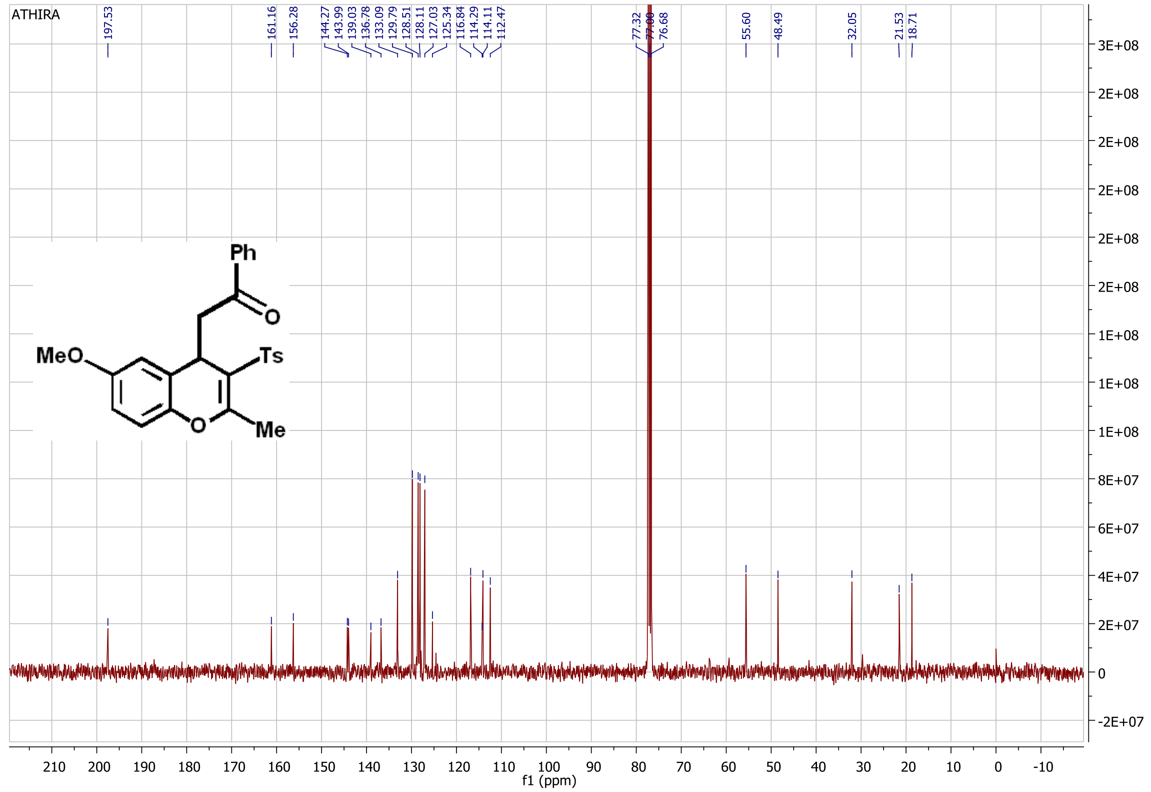

^{1}H NMR spectrum of **8ga** (500 MHz, $CDCl_3$)

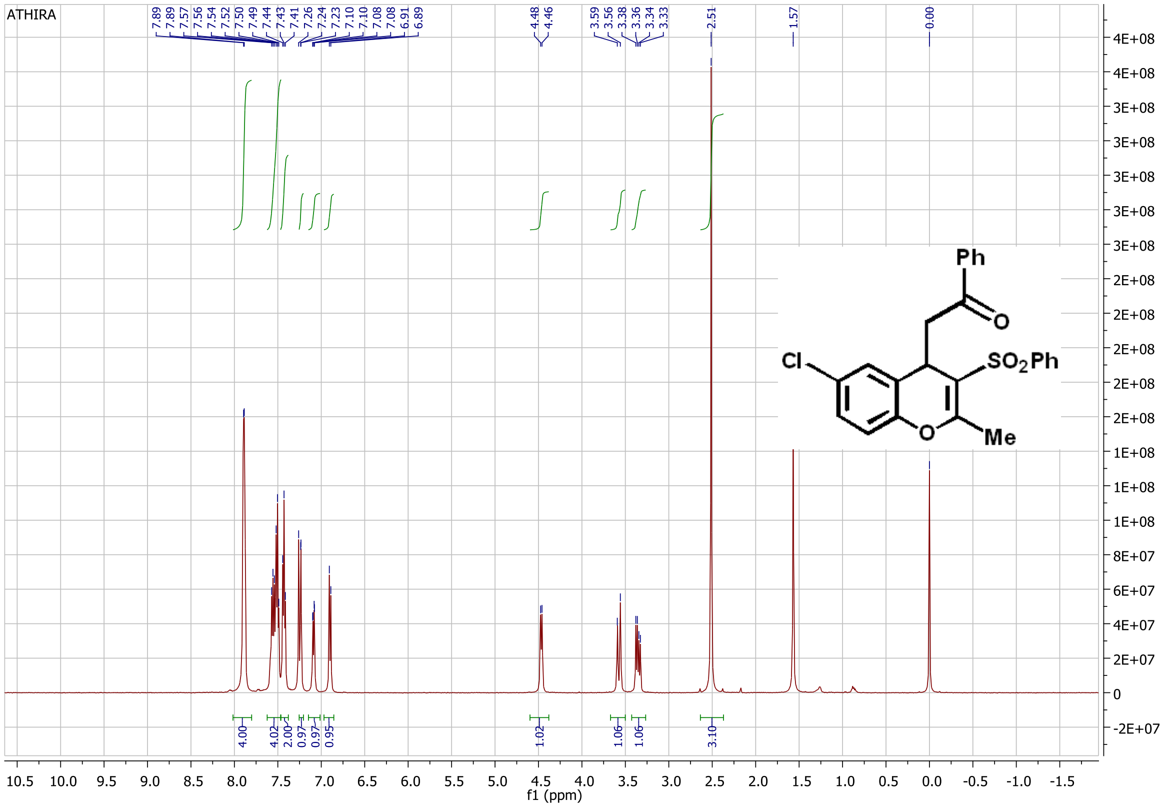

^{13}C NMR spectrum of **8ga** (75 MHz, $CDCl_3$)

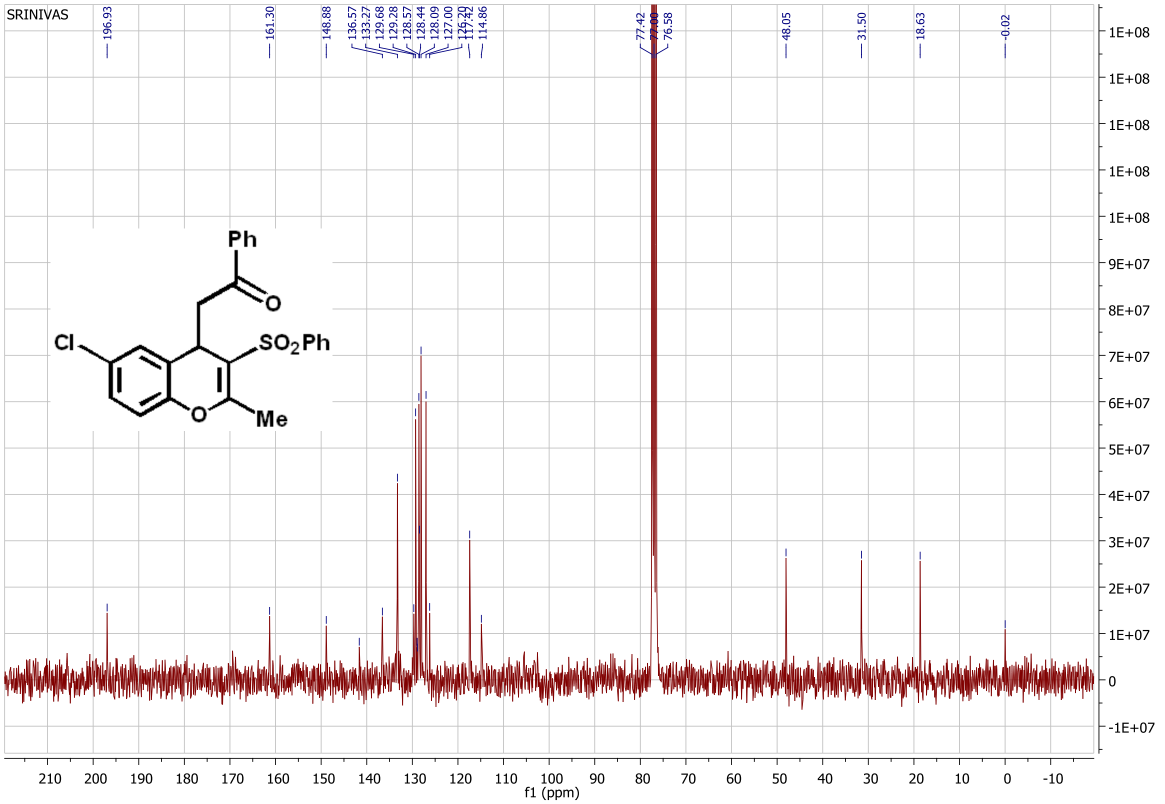

^{1}H NMR spectrum of **8gb** (500 MHz, $CDCl_3$)

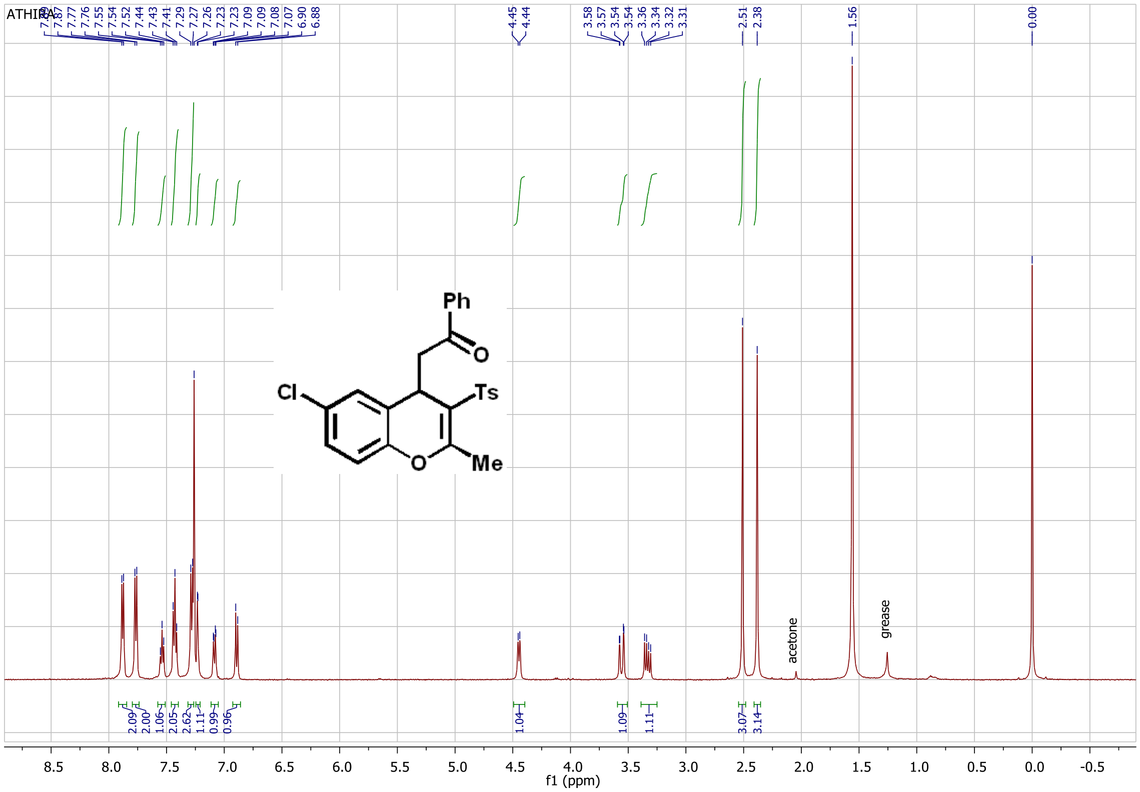

^{13}C NMR spectrum of **8gb** (100 MHz, $CDCl_3$)

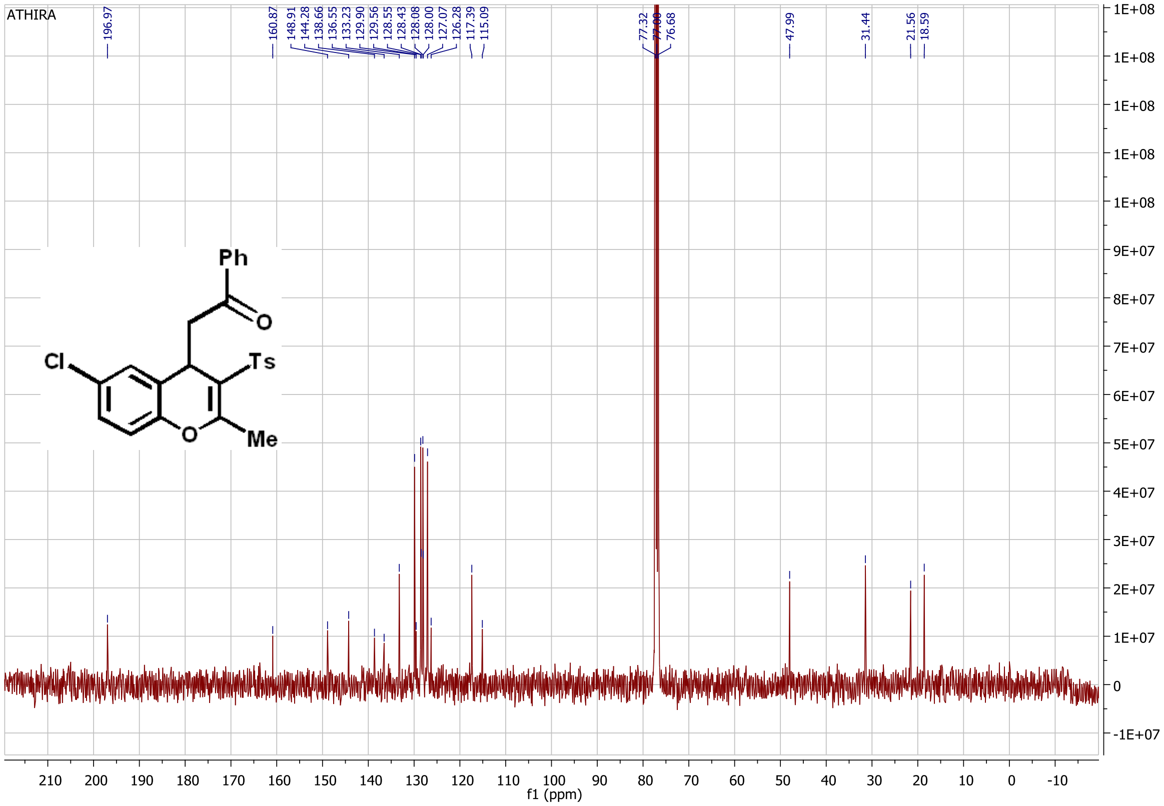

^{1}H NMR spectrum of **8ha** (500 MHz, $CDCl_3$)

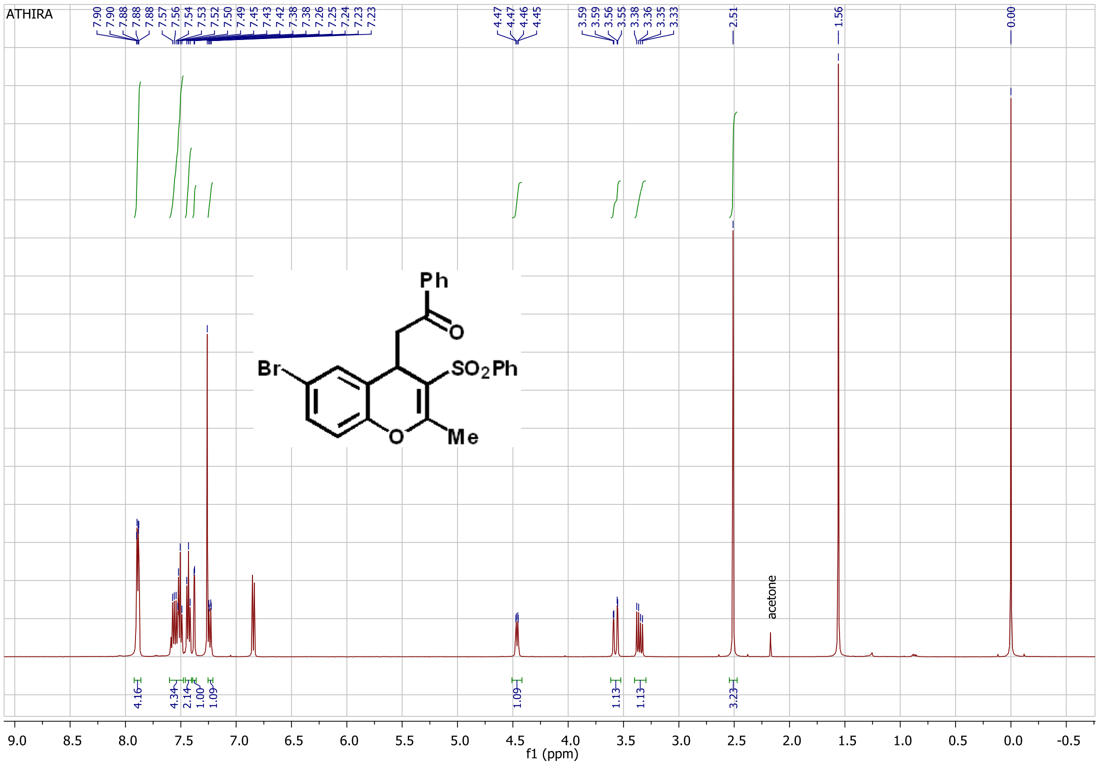

^{13}C NMR spectrum of **8ha** (125 MHz, $CDCl_3$)

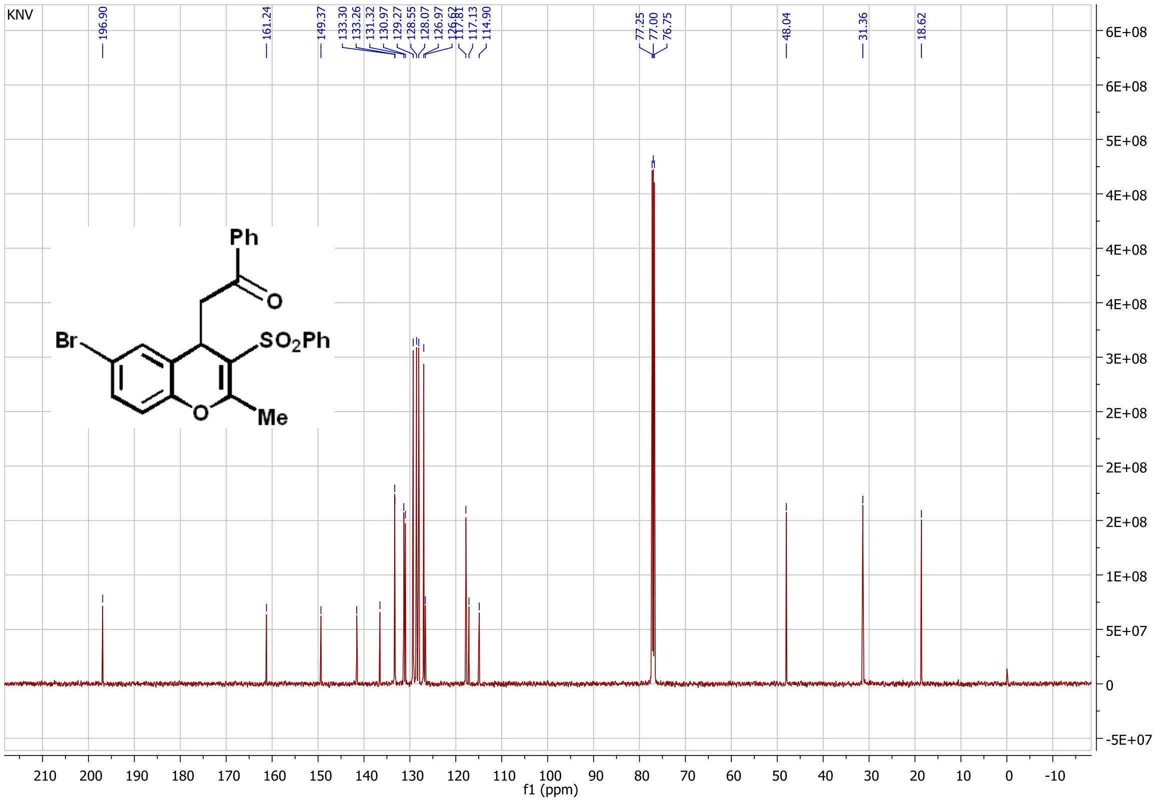

^{1}H NMR spectrum of **8hb** (400 MHz, $CDCl_3$)

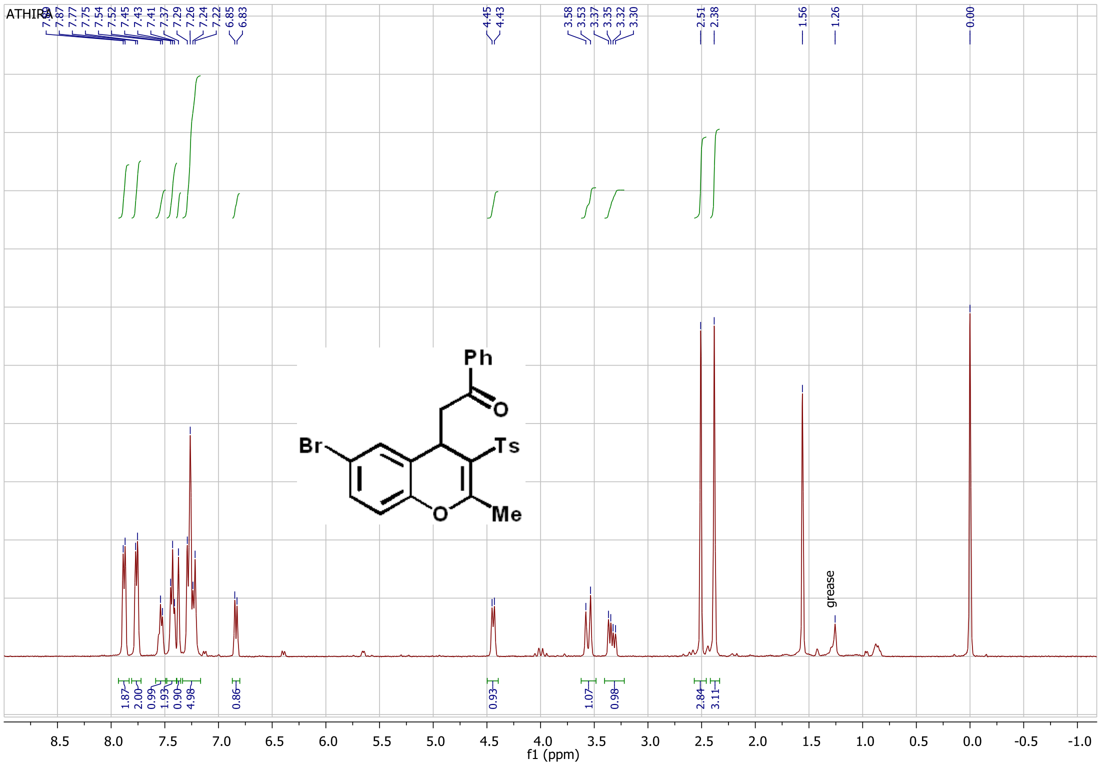

^{13}C NMR spectrum of **8hb** (100 MHz, $CDCl_3$)

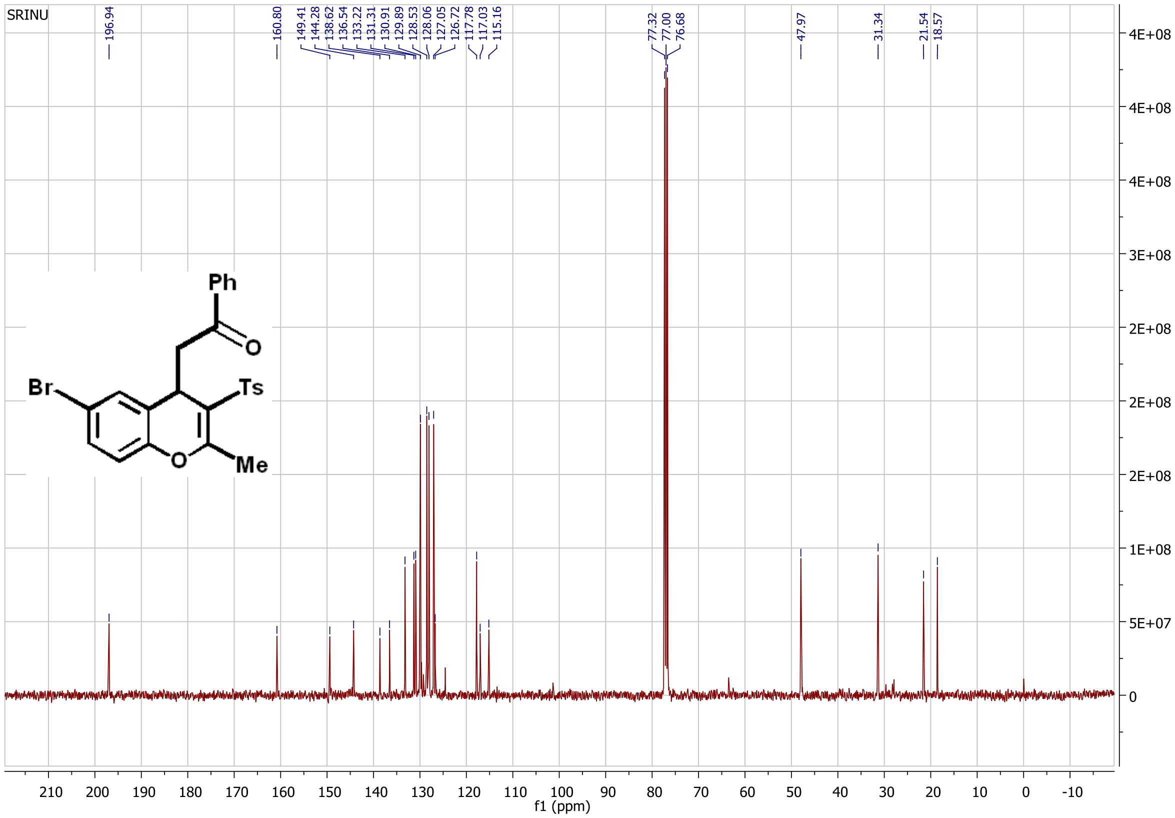

^{1}H NMR spectrum of **8ia** (400 MHz, $CDCl_3$)

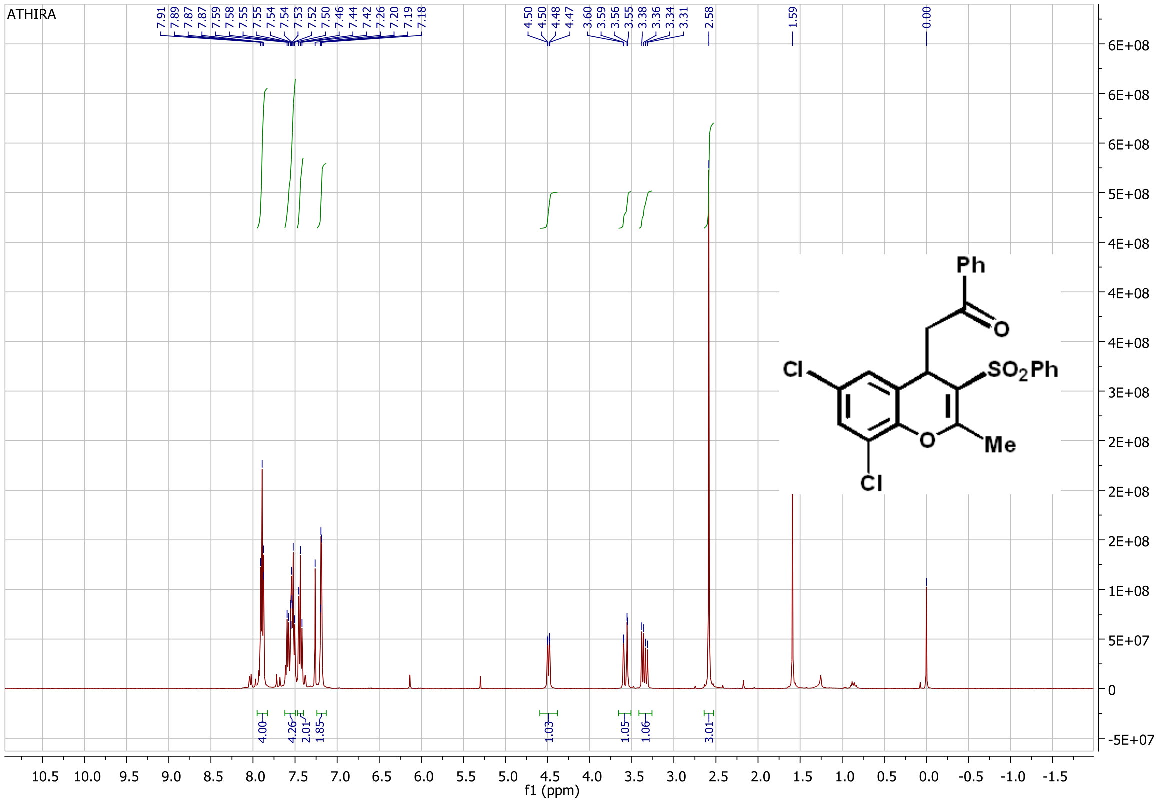

^{13}C NMR spectrum of **8ia** (100 MHz, $CDCl_3$)

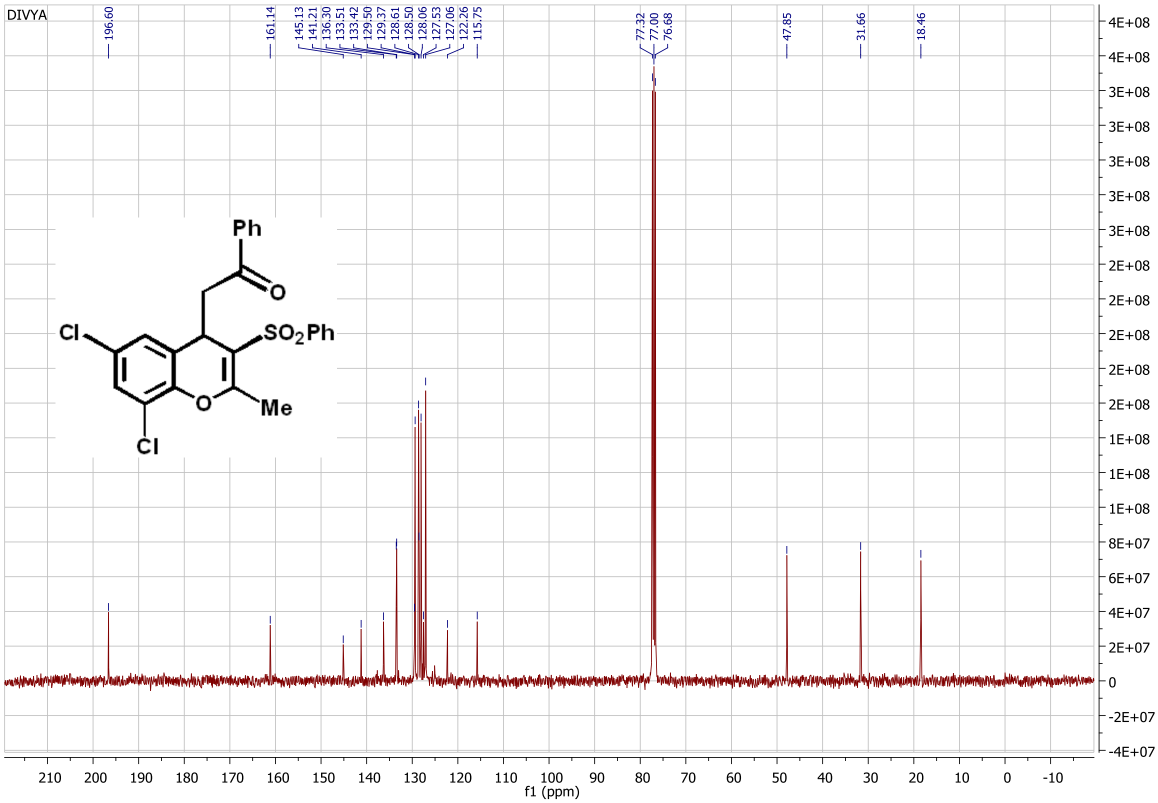

^{1}H NMR spectrum of **8ib** (500 MHz, $CDCl_3$)

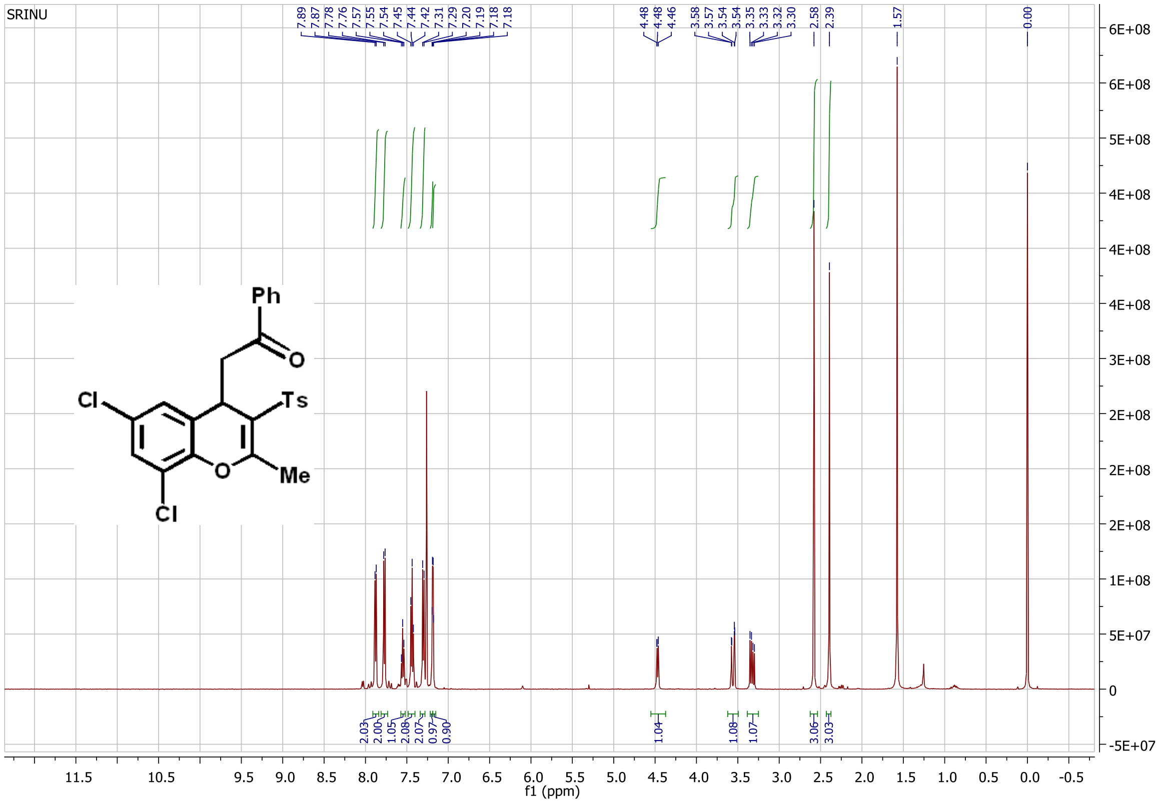

^{13}C NMR spectrum of **8ib** (100 MHz, $CDCl_3$)

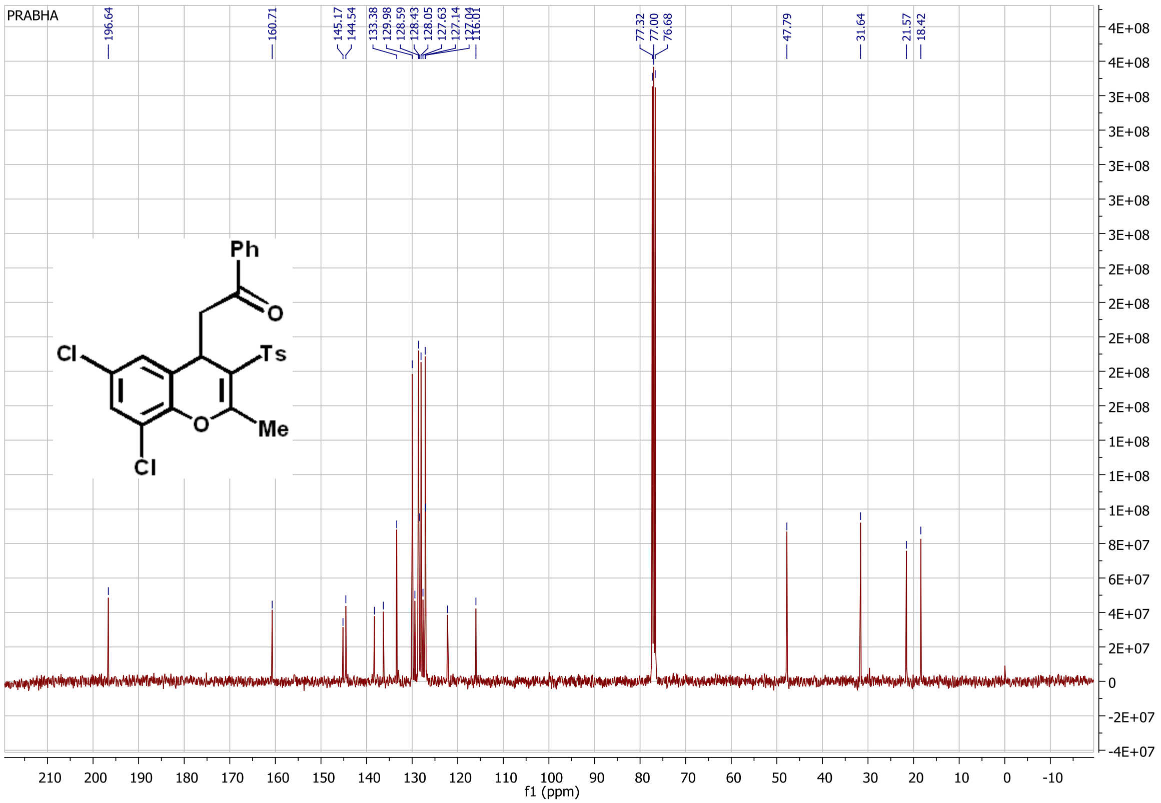

Supplement: File 1 — Experimental part and NMR spectra of synthesized compounds. [file Beilstein_J_Org_Chem-12-16-s001.pdf]
